# New Insights Regarding the Use of Relevant Synthetic Compounds in Dentistry

**DOI:** 10.3390/molecules29163802

**Published:** 2024-08-10

**Authors:** Stefania-Irina Dumitrel, Anamaria Matichescu, Stefania Dinu, Roxana Buzatu, Ramona Popovici, Dorin Cristian Dinu, Dana Cristina Bratu

**Affiliations:** 1 Department of Toxicology, Drug Industry, Management and Legislation, Faculty of Pharmacy, Victor Babes University of Medicine and Pharmacy, 2nd Eftimie Murgu Sq., 300041 Timisoara, Romania; stefania-irina.dumitrel@umft.ro; 2 Department of Preventive, Community Dentistry and Oral Health, Faculty of Dental Medicine, Victor Babes University of Medicine and Pharmacy, 14A Tudor Vladimirescu Ave., 300173 Timisoara, Romania; 3 Translational and Experimental Clinical Research Centre in Oral Health, Victor Babes University of Medicine and Pharmacy, 14A Tudor Vladimirescu Ave., 300173 Timisoara, Romania; 4 Department of Pedodontics, Faculty of Dental Medicine, Victor Babes University of Medicine and Pharmacy, 9 No., Revolutiei 1989 Bv., 300041 Timisoara, Romania; dinu.stefania@umft.ro; 5 Pediatric Dentistry Research Center, Faculty of Dental Medicine, Victor Babes University of Medicine and Pharmacy, 9 No., Revolutiei 1989 Bv., 300041 Timisoara, Romania; 6 Department of Dental Aesthetics, Faculty of Dental Medicine, Victor Babes University of Medicine and Pharmacy, 9 No., Revolutiei 1989 Bv., 300041 Timisoara, Romania; drbuzaturoxana@gmail.com; 7 Department of Management, Legislation and Communication in Dentistry, Faculty of Dental Medicine, Victor Babes University of Medicine and Pharmacy, 9 No., Revolutiei 1989 Bv., 300041 Timisoara, Romania; ramona.popovici@umft.ro; 8 Family Dental Clinic, Private Practice, 24 Budapesta Street, 307160 Dumbravita, Romania; dorin@dr-dinu.com; 9 Department of Orthodontics II, Faculty of Dental Medicine, Victor Babes University of Medicine and Pharmacy Timisoara, 9 No., Revolutiei 1989 Bv., 300041 Timisoara, Romania; bratu.cristina@umft.ro

**Keywords:** antiseptics, cetylpyridinium, chlorhexidine, dentistry, hydrogen peroxide, methods, octenidine, povidone-iodine, sodium hypochlorite, synthetic drugs

## Abstract

Worldwide, synthetic compounds are used for both in-office and at-home dental care. They are a valuable resource for both prophylactic and curative treatments for various dental problems, such as tooth decay, periodontal diseases, and many more. They are typically preferred due to their broad range of actions and ability to produce targeted, rapid, and long-lasting effects. Using a 0.12% chlorhexidine mouthwash is capable of reducing the plaque index from 47.69% to 2.37% and the bleeding index from 32.93% to 6.28% after just 2 weeks. Mouthwash with 0.1% OCT is also highly effective, as it significantly lowered the median plaque index and salivary bacterial counts in 152 patients in 5 days compared to a control group (*p* < 0.0001), while also reducing the gingival index (*p* < 0.001). When povidone-iodine was used as an irrigant during the surgical removal of mandibular third molars in 105 patients, it resulted in notably lower pain scores after 2 days compared to a control group (4.57 ± 0.60 vs. 5.71 ± 0.45). Sodium hypochlorite is excellent for root canal disinfection, as irrigating with 1% NaOCl completely eliminated the bacteria from canals in 65% patients. A 0.05% CPC mouthwash proved effective for perioperative patient care, significantly decreasing gingival bleeding (*p* < 0.001) and suppressing *Streptococcus* levels even one week post-surgery. Lastly, a 6% H2O2 paint-on varnish and 6% H2O2 tray formulations successfully bleached the teeth of 40 patients, maintaining a noticeably whiter appearance up to the 6-month follow-up, with significant color differences from the baseline (*p* < 0.005). Synthetic compounds have a large research base, which also provides a greater awareness of their mechanism of action and potential adverse effects. For a better understanding of how they work, several methods and assays are performed. These are protocolary techniques through which a compound’s efficacy and toxicity are established.

## 1. Introduction

Unfortunately, oral health is sometimes a privilege instead of a right, leading to physiological and psychological imbalances in the general population. The World Health Organization included oral health in the top 10 human health standards, as disruptions at this level could impact people’s quality of life. Dental problems represent a major concern, especially among older people, who are more vulnerable, and people from low- or middle-income countries. Sometimes, oral diseases have systemic consequences, because they can lead to heart disease, diabetes, dementia, infections, and, in the worst cases, even cancer. Mental state is also a relevant concern, because it can impact people’s general appearance, since dental problems, such as dental caries and periodontal disease, can further result in tooth loss [1,2]. The Global Burden of Disease reported in 2019 that oral diseases, such as dental caries, periodontitis, and tooth loss, affect more than 44.5% of people worldwide, making it a priority in terms of the health of the general population [2].

The oral cavity harbors many microbes, including bacteria, fungi, and viruses, that are fundamental for maintaining the optimal oral health. These microorganisms can result in several dental issues under various circumstances, such as tooth decay, gingivitis, periodontitis, and even oral cancer [3,4].

Synthetic compounds remain the gold standard in dental care because of their wide spectrum of activity and durable effects. The most known and used antiseptic in the dentistry field is chlorhexidine. However, other chemicals, including octenidine, cetylpyridinium chloride, povidone-iodine, and sodium hypochlorite, are also of significant importance due to their versatile applications. Hydrogen peroxide is another popular compound used in dentistry, which is well known for its bleaching properties that enhance dental aesthetics and boost patients’ self-esteem. Although these compounds present rapid effects with prolonged activity, they come with certain risks [5,6].

Experimental methods and assays are essential not only in dentistry, but in all medical fields, for several reasons. They help to minimize adverse effects by allowing researchers to observe and determine the mechanisms of action of compounds. This process provides valuable information about the safety and effectiveness of treatments, materials, and techniques. Additionally, these methods help to identify the variability in individual susceptibilities to diseases, which optimizes suitable, individualized treatments for each person’s needs. In a nutshell, these methods offer crucial insights into the key components of optimal oral health, thereby enhancing the quality of dental care for patients [7,8,9,10].

## 2. Synthetic Compounds Used in Dentistry

### 2.1. Chlorhexidine (CHX)

#### 2.1.1. History

**Figure 1:**
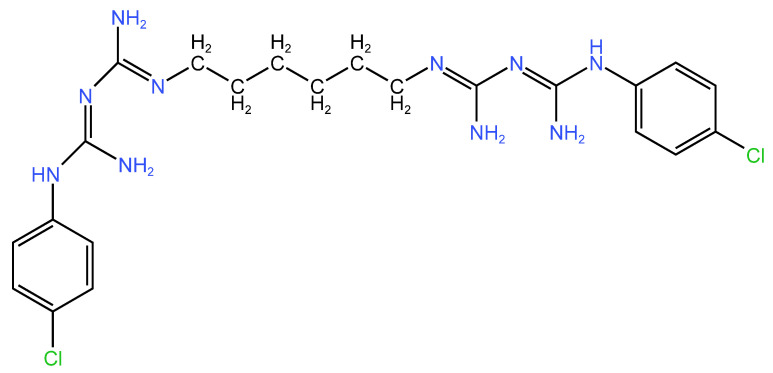
The molecular structure of chlorhexidine.

CHX (Figure 1) is a biguanide compound that has been in therapeutic usage as an antiseptic since 1950 in the UK. In 1972, Löe and Schiot presented the first evidence supporting its beneficial role in stomatology [11]. They demonstrated CHX’s efficacy in preventing dental caries and reducing dental plaque. Since then, several dental applications have used chlorhexidine for its disinfectant properties, including halitosis reduction, periodontal disease treatment, implant surgery, root canal treatment, peri-implant mucositis, and oral mucositis. Due to its high probability of resulting in adverse effects, some formulations have been tried to mitigate them. When using CHX, one of the most common problems is tooth staining. For this problem, some anti-discoloration systems such as ascorbic acid, sodium metabisulphite, and sodium perborate monohydrate have been added to CHX products, and it has been demonstrated that they reduce the incidence of tooth staining without altering CHX’s efficiency. Another unpleasant outcome after using CHX products may be due to the alcohol content. For this reason, alcohol-free mouthwashes are now in trend, because they are just as effective as alcohol-containing mouthwashes while causing fewer adverse effects [12,13,14,15,16].

Although chlorhexidine is effective in reducing plaque and biofilm formation, sometimes, its use has been associated with dysbiosis. For this reason, further investigation is needed to develop safer and more targeted oral care products that are efficient without targeting commensal microorganisms. Another important issue to be addressed is the risk of resistance of oral bacteria against chlorhexidine. Combination therapies and innovative delivery systems, such as slow-release formulations, seem promising options that could sustain antimicrobial effects over a longer period, reducing the need for frequent application and potentially decreasing resistance and other side effects. Such strategies represent a viable area for future research, aiming to optimize the benefits of CHX while addressing its limitations [17,18,19].

#### 2.1.2. Chemical Structure

This cationic molecule is composed of two 4-chlorophenyl rings and two biguanide groups that are connected by a core hexamethylene chain [20]. Its structure is crucial in its mechanism of action, which involves interfering with membrane functions due to cationic groups (nitrogen atoms) and causing cellular disruption by binding to the negatively charged cell wall, killing bacteria. This process provides therapeutic benefits for pathologies such as caries prevention, gingivitis, and periodontal diseases [21].

#### 2.1.3. Antimicrobial Spectrum and Mechanism of Action

It is currently used as an antimicrobial and antiseptic agent due to its broad spectrum and ability to interfere with membrane permeability and cause cellular disruption [21]. CHX results in long-lasting antiseptic adherence to both hard and soft oral tissue. Hence, the antiseptic slowly releases at a potent dosage, maintaining its antimicrobial impact [22].

It exhibits a wide range of antibacterial effects, targeting both Gram-positive (*Streptococcus mutans*, *Lactobacillus*, *Enterococcus faecalis*, *Streptococcus viridans*, and *Streptococcus salivarius*) and Gram-negative bacteria (*Porphyrmonas gingivalis*, *Fusobacterium nucleatum*, *Prevotella* spp., *Treponema denticola*, *Pseudomonas aeruginosa*, *Escherichia coli*, *Klebsiella pneumoniae*, and *Acinetobacter baumannii*) as well as yeasts (*Candida* spp. and *Cryptoccocus neoformans*) dermatophytes, and certain viruses (HSV and HPV) [21,23,24,25].

**Figure 2:**
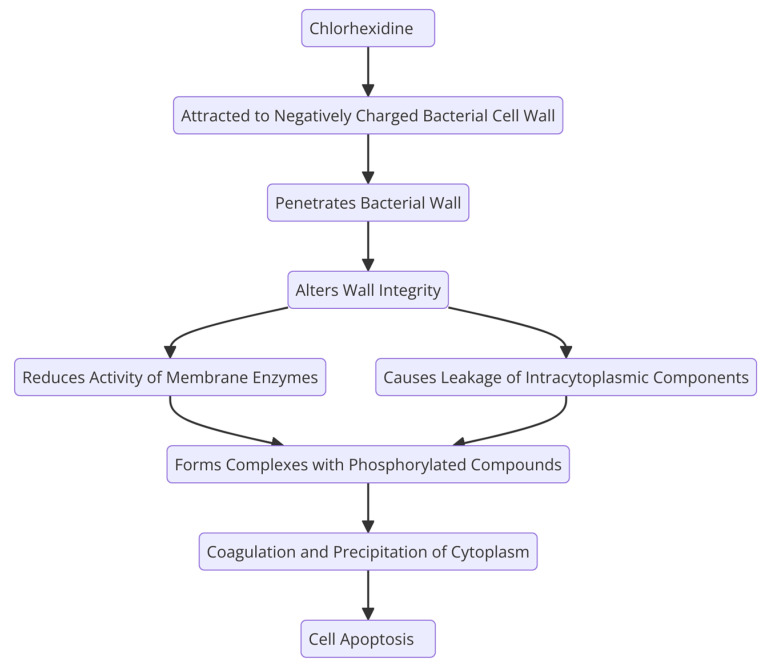
The mechanism of action of chlorhexidine.

CHX’s effect is time- and dose-dependent. It has bacteriostatic properties at low concentrations (it prevents bacterial growth) and bactericidal effects at high doses (it kills bacteria at this stage) [26]. Even at low concentrations, CHX alters the bacterial osmotic balance. Chlorhexidine mouthwashes at concentrations of 0.12% are most commonly prescribed for plaque reduction and after periodontal and implant surgery because of their low incidence of adverse effects. This concentration, even when used twice daily for a prolonged period, does not negatively affect the oral microbial flora. Using CHX at a concentration of 0.2% increases the risk of side effects, resulting in more cases of tooth discoloration and taste problems. However, its effectiveness in removing plaque is comparable to that of the 0.12% concentration [27,28]. Because it is a cationic molecule, it is strongly attracted to the negatively charged bacterial cell wall. In this manner, it penetrates the bacterial wall and alters its integrity. This causes the leakage of some intracytoplasmic components, as well as a reduction in the activity of some cytoplasmic membrane enzymes. In the final stage, it forms complexes with phosphorylated compounds, resulting in the coagulation and precipitation of the cytoplasm, disrupting cellular function and leading to cell apoptosis (Figure 2) [29]. 

#### 2.1.4. Formulations and Current Uses of CHX in Dentistry

Chlorhexidine is available in various oral preparations, including mouthwashes, toothpaste, gels, lozenges, sprays, chips, and varnishes [11,29,30]. These products contain different concentrations based on the formulation: mouthwashes (0.1 to 0.2%), toothpaste 0.12%, gels (1% to 2%), sprays (0.1–0.2%), varnishes (0.1% to 40%), and chips, which usually contain 2.5 mg of CHX [11,30,31,32]. CHX mouthwashes at 0.12 and 0.2% concentrations appear to be the most effective formulations compared to gels and dentifrices in several dental applications because of their potency in inhibiting plaque formation, as well as their good patient compliance and ease of application. Studies have demonstrated that these concentrations effectively maintain good oral hygiene due to their anti-plaque effects. Additionally, CHX mouthwashes showed the best results when used prophylactically before surgery to prevent oral biofilm formation and promote wound healing, thereby avoiding post-surgical complications. Rinsing with CHX mouthwash at both concentrations reduced clinical signs of gingivitis, as well as the disease’s prevalence compared to gels. CHX mouthwash was also better than the gel at combating aphthous ulceration, as it reduced the incidence of ulcerations compared to the gel, which did not have any effect on the recurrence of the disease [12,21,27,33,34,35,36,37,38]. However, for patients with stage I–III periodontitis, peri-implant mucositis, and peri-implantitis with low accessibility in deep pockets, 2.5 CHX chips are the preferred option due to their sustained release [35,39,40,41,42,43].

Chlorhexidine is preferred by dentists and the general public for its efficacy in combating oral infections and maintaining dental hygiene. It is used for dental caries prevention because of its capacity to reduce *Streptococcus* spp., *Lactobacillus* spp., and dental plaque accumulation, which are known to be potential risk factors that can contribute to cavity formation [44,45]. However, its effectiveness in caries prevention is still under discussion [35]. The presence of microbial biofilms in or near the gingival sulcus can cause gingival tissue inflammation, which can potentially progress to periodontitis and tooth loss [46,47,48]. Multiple studies have demonstrated CHX’s beneficial action against gingivitis and periodontitis through the inhibition of cytokines, *Streptococcus* spp., *P. gingivalis*, *F. nucleatum,* and *A. actinomycetemcomitans,* as well as its ability to reduce plaque buildup and prevent biofilm formation [49,50,51,52,53,54]. Scientific evidence proves CHX’s efficacy against *E. faecalis*, a frequently encountered bacterium in root canal infections [55]. Hence, the antibacterial activity of CHX makes it an excellent candidate as a root canal irrigator [54,56]. Moreover, a recent study showed its beneficial effect in treating chemotherapy-induced oral mucositis [15]. The evidence supporting CHX usage is presented in Table 1. 

#### 2.1.5. Adverse Effects

CHX’s adverse reactions include xerostomia (a lack of moisture in the mouth), which can lead to major oral issues, because saliva plays a crucial role in maintaining the optimal oral health [21,61,62]. Insufficient saliva production leads to several challenges, such as speech difficulties, problems with mastication, tooth decay, difficulty using dental prostheses, frequent infections (candidiasis), halitosis, the degeneration of soft tissues, and an overall a decrease in quality of life [63]. It can also cause calculus formation, which is linked to the development of caries, gingivitis, and periodontitis [64,65]. Other frequent local side effects are brown staining of the teeth, restorative materials, and the back of the tongue [66,67]. Alcohol-containing mouthwashes with 0.12% and 0.2% CHX caused extrinsic tooth staining in participants (*p* < 0.05) [68]. In a study conducted on 27 participants who used a 0.12% chlorhexidine mouthrinse, all of the subjects reported taste disturbances, while 70% had tongue numbness, 18.5% mild irritation, 2 patients dropped out because of the gingival desquamation and burning sensation (the symptoms were transient, disappearing after 3 days without treatment), and 2 reported parotid gland discomfort and parotitis (the discomfort stopped after avoiding the CHX-containing products) [69]. Another study conducted on 40 participants confirmed that chlorhexidine mouthrinse at 0.12% caused taste disturbance in 85.4%, xerostomia in 78.1%, and tooth discoloration in 58.6% of patients [70]. Oral usage of CHX can lead to potentially serious adverse effects (even in the dentistry field), including Type I and Type IV hypersensitivity reactions accompanied by severe anaphylaxis [71,72]. A case report described a 69-year-old woman who suffered an allergic reaction (an erythematous patch on her back) after using a 0.2% CHX mouthwash. She stopped using CHX-based products and no further skin reactions developed [73]. Furthermore, there have been reports of severe allergic reactions following the use of CHX mouthwash, leading to respiratory arrest and even fatalities [21]. Three children experienced anaphylaxis after using CHX mouthwash and antiseptic tablets [74]. A 21-year-old female suffered an anaphylactic reaction after using a CHX mouthwash that manifested through tachycardia, throat tightness, chest congestion, and itchiness, and in the end, she lost consciousness [75].

Antimicrobial resistance is another major concern, because it reduces CHX’s effectiveness. If CHX is used excessively, it can lead to resistance [76]. Mechanisms that cause this resistance include multidrug efflux pumps, genetic changes, and alterations in the cell membrane. These mechanisms facilitate the development of antibiotic resistance in other bacteria, particularly those that are resistant to multiple drugs [19]. There have been reports of bacteria with a decreased ability to respond to chlorhexidine. Gram-positive bacteria can develop resistance by enzymatically breaking down antibiotics through the production of beta-lactamase. Another mechanism is represented by reducing the effectiveness of their target site, the penicillin-binding protein, by acquiring external DNA or altering protein genes [19]. Gram-negative bacteria mostly develop resistance to chlorhexidine through the use of multidrug efflux pumps [76,77].

#### 2.1.6. Experimental Methods Used to Evaluate the Efficacy and Safety of CHX in Dental Applications

Mensitieri et al. [78] analyzed the antibacterial activity of chlorhexidine gluconate (CHG) against *S. mutans* by itself and in three different mouthwash formulations (f): f1-mouthwash with CHG without natural herbs, and f2- and f3-mouthwashes containing natural herbs. The microbroth dilution method was used to determine the MIC (minimum inhibitory concentration) and MBC (minimum bactericidal concentration). The microbroth dilution technique involved serial dilutions of the tested substances in broth culture followed by incubation with *S. mutans* to identify the lowest concentrations that inhibited visible bacterial growth (MIC) and killed the bacteria (MBC). The most potent were f1 and CHG at an MIC of 1.25 µg/mL and an MBC of 15 µg/mL. The BPC (biofilm prevention concentration) was obtained after crystal violet (CV) staining to assess the residual biofilm percentage after 24 h of treatment. The crystal violet assay involves treating biofilms with the tested formulations, staining them with crystal violet dye, removing the excess dye with a solubilizer (ethanol in this case), and quantifying the residual biofilm mass by measuring the absorbance at 540 λ. The best results were obtained from CHG and f1 at 2.5 µg/mL compared to f2 at 5 µg/mL and f3 at 10 µg/mL. Both f1 and CHG presented the best inhibitory effects on mature biofilms at their undiluted concentrations (2 mg/mL), reducing the biofilm’s matrix by 50% at 60 µg/mL. After an MTT assay, the cell viability of *S. mutans* in the mature biofilm was evaluated after 24 h of incubation. The MTT assay (tetrazolium dye assay) was conducted by using MTT solution to stain the culture cells, followed by a solubilizing agent (DMSO) to dissolve the tetrazolium salts. Afterward, the plates were incubated in the dark for 40 min and the absorbance was read at 570 λ. CHG and f1 revealed significant reductions in cell viability, even at a concentration of 30 µg/mL, and an almost complete reduction at 120 µg/mL. The other two formulations required at least double these concentrations to achieve the same results. MTT and CV assays were also used to evaluate the integrity of the mature biofilm and the viability of the cells after 5 min of treatment. CHG and f1 reduced the cell viability by 50% at their undiluted concentrations (2 mg/mL) in the mature biofilm, but did not have a significant effect on decreasing the total biofilm biomass.

The cytotoxic effect of 1% CHX on human keratinocytes was tested by analyzing histological changes and surface morphology. The results were established after staining with hematoxylin–eosin and using electron microscopy. It was shown that, after 15 min of exposure, less than 20% of the keratinocytes survived, indicating a high cytotoxic effect on the cell line [79]. 

In an in vitro study, the effect of CHX at concentrations of 0.002%, 0.02%, 0.2%, and 2% on human osteoblasts, myoblasts, and fibroblasts was analyzed after 1, 2, and 3 min of exposure. To assess cell viability and migration, a Cell Counting Kit and a scratch test, respectively, were performed. The results revealed that, even at the lowest concentration (0.002%), both the cell survival rate and cell migration were affected after 2 and 3 min of exposure. After 24 h, it was observed that there were open scratch defects after using ≥0.02% at all time stamps. Additionally, the concentration of 2% was demonstrated to be highly cytotoxic for any exposure duration [80]. According to another in vitro study using the MTT assay, the viability of fibroblastic cells was reduced after 1 min of exposure to 0.006% (*v/v*) CHX. After staining with Annexin V to measure the apoptotic cell levels and with propidium iodide to quantify the necrotic cells, it was observed that CHX decreased the fibroblasts’ proliferative capacity and dramatically enhanced their necrosis and apoptosis rates compared to a control group. Also, there was a significant decrease in the percentage of wound closure in a time-dependent manner, as observed after performing wound healing assay at 0, 12, and 24 h [81]. 

An in vivo study was conducted to determine CHX’s effects in periodontitis treatment. For a week, ligature-induced periodontitis was generated on rat molars. The animals were killed post-treatment after scaling and root planing (SRP) + 0.12% CHX, SRP+ irrigation with 0.12% CHX, SRP + 0.2% CHX, and SRP+ irrigation with 0.2% CHX. Histometric analyses were performed to analyze the extent of bone loss or regeneration. Immunochemical staining was used to identify the specific proteins involved in the inflammatory and metabolic processes. These methods were beneficial in determining the positive effects of using CHX after SRP on protecting periodontal tissue and reducing inflammation [82].

#### 2.1.7. Clinical Trials

Sreenivasan et al. [44] demonstrated that chlorhexidine decreased the total number of occurrences of high gingival index and dental plaque scores in 45 patients after 2 weeks of treatment with a 0.12% mouthwash used twice a day. It was shown that CHX reduced gingivitis by 15% and plaque by 19% after 1 week, and by 25% and 35%, respectively, after 2 weeks. Furthermore, CHX decreased oral polymorphonuclear leukocytes by 35.9% after 7 days and by 54.9% after 14 days. These results were confirmed in another study conducted by Ripari et al. [83], in which 0.12% CHX mouthwash was tested in 21 subjects. The treatment was applied twice daily for 30 s for two weeks. The plaque index and bleeding index were significantly reduced from 47.69% to 2.37% and from 32.93% to 6.28%, respectively, at the end of the trial. However, 12 patients complained about the unpleasant taste and burning sensation. In another study, chlorhexidine was tested in 33 patients in two combinations to assess its role in early periodontal wound healing. They were divided into three equal groups: Group 1 (received CHX+Anti-Discoloration system+Hyaluronic Acid), Group 2 (CHX+Anti-Discoloration system), and Group 3 (control). They applied the treatment for 60 s twice daily for 14 days. After 3 days, Group 1 showed a significant decrease in the Early Healing Index, completely healing 72.73% of the patients, compared to 36.37% in Group 2 and 27.27% in the control group. By the end of the trial, in Group 1, 90% of the subjects had completely healed, and in Group 2, 63.64% had. These results suggest that combined therapies with chlorhexidine may increase their effectiveness in promoting wound healing after periodontal disease [84]. A 0.2% chlorhexidine mouthwash was tested in 38 patients for 2 weeks, either with the sole compound or in combination with an anti-discoloration system (AD) to determine its potential for treating stage III and IV periodontitis. Two groups of 17 patients each rinsed with mouthwash for 1 min. The CHX+AD group showed the best results after 14 days, with the highest reduction in plaque index (*p* = 0.02) and gingival index (*p* = 0.01) scores compared to the control. In the CHX group, more patients reported teeth and tongue stains, as well as oral discomfort [85].

### 2.2. Octenidine (OCT)

#### 2.2.1. History

**Figure 3:**
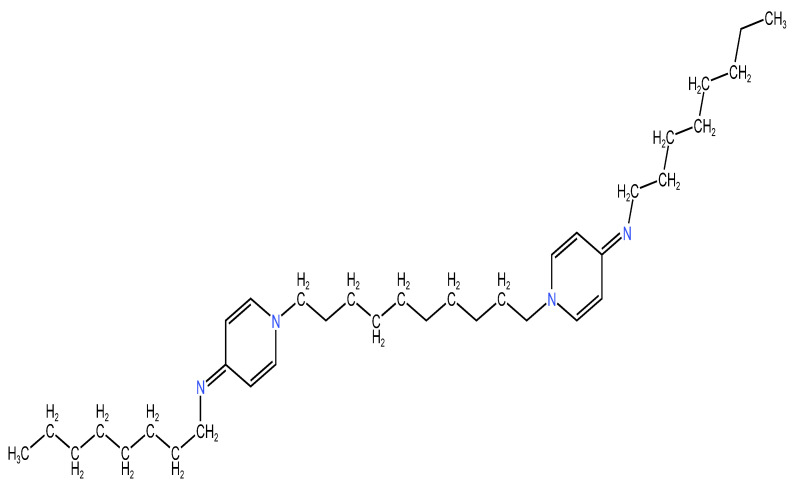
The molecular structure of octenidine.

Octenidine (Figure 3) dihydrochloride (OCT) is an antibacterial cationic molecule that was created at the Sterling-Winthrop Research Institute in Rensselaer, NY, during the 1980s [86]. It is an antiseptic that belongs to the bispyridine family [87]. Today, octenidine is used in dentistry for various purposes, including dental caries prevention, gingivitis, periodontitis, the remineralization of enamel and dentin, and root canal treatment. However, while beneficial, octenidine use can cause adverse effects. The most frequent side effects are tooth staining and buccal tissue irritation. Other possible adverse effects are burning sensations, taste disturbances, tongue numbness, swelling, and hives. To minimize the risk of these side effects and ensure sustained drug release, newer delivery systems have been developed. These include nanoparticles composed of mesoporous silica, nanocellulose wound dressings, combinations of nanocellulose with amphiphilic copolymers containing, poly(lactic-acid)-based nanoparticles, octenidine, and poly(glycolic acid)-based biodegradable sutures [86,88,89,90,91,92]. Octenidine is generally considered safe, however, there is a lack of comprehensive information on its long-term effects for use in the oral cavity. For this reason, more studies should focus on how prolonged exposure could interact with the mouth tissue and cause adverse reactions. Additionally, more targeted research is needed on octenidine’s potential in treating periodontitis, as it has a prevalence from 20 to 50% worldwide [86,93,94,95,96].

#### 2.2.2. Chemical Structure

Octenidine is a cationic biguanide surfactant compound that shares a similar structure to chlorhexidine [86,97]. OCT has a reduced toxicity compared to other guanides like chlorhexidine because of the absence of an amide and ester structure, eliminating the potential formation of metabolites and making it a safer option in dental treatments. Ten methylene groups separate the two cationic pyridines. Each aminopyridine contains a terminal hydrophobic octanyl group. The cationic parts are particularly important, because they offer octenidin’s therapeutic benefits in the dentistry field [98]. 

#### 2.2.3. Antimicrobial Spectrum and Mechanism of Action

It combats both Gram-positive (*Streptococcus mutans*, *Lactobacillus* spp., *Staphylococcus epidermidis*, *Staphylococcus aureus*, methicillin-resistant *Staphylococcus aureus*, and *Streptococcus pyogenes*) and Gram-negative (*Aggregatibacter actinomycetemcomitans*, *Porphyromonas gingivalis*, *Prevotella intermedia*, *Pseudomonas aeruginosa*, *Klebsiella pneumoniae*, *Escherichia coli*, and *Chlamydia trachomatis*) bacteria, mycoplasma, and fungi (*Candida albicans*, *Candida glabrata*, *Candida auris*, *Candida parapsilosis*, and *Candida tropicalis*) [87,99]. It also exhibits antiviral properties against SARS-CoV-2 and HSV-1 [100,101,102,103]. Octenidine mouthwash at a concentration of 0.1% is the most used formulation, because it has proven efficient in several dental applications with minimal adverse effects. At this concentration, it possesses bactericidal activity against *S*. *mutans*, *Lactobacillus* spp., and *P*. *intermedia*. It was observed that raising its dosage to 0.15% or 0.2% resulted in more frequent unpleasant outcomes [86].

**Figure 4:**
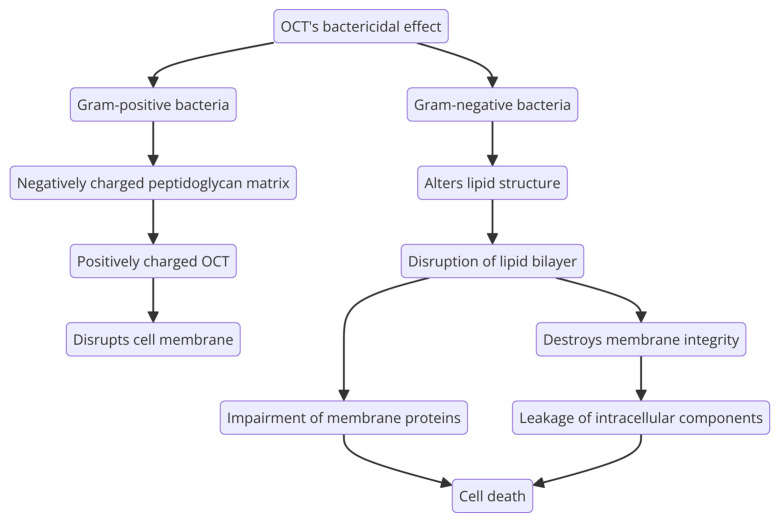
The mechanism of action of octenidine.

The underlying mechanism of OCT’s bactericidal effect (Figure 4) is due to the alterations it causes in the structure of the lipids in the outer membrane of Gram-negative bacteria. A disruption in the lipid bilayer destroys membrane integrity because of changes in fluidity and depolarization, resulting in the leakage of intracellular components and cell death [98]. At the same time, the functionality and integrity of the membrane’s proteins are impaired because the positively charged groups of OCT interact with the negatively charged groups of the membrane’s lipids [104]. Gram-positive bacteria lack an outer membrane made of phospholipids and lipopolysaccharides. Instead, they have a thicker peptidoglycan matrix containing negatively charged cell wall components, which attract the cationic antiseptic that disrupts the cell membrane [105].

#### 2.2.4. Formulations and Current Uses of OCT in Dentistry

Octenidine is found in different formulations such as mouthrinses (0.1%), wound gels (0.05% and 0.1%), wound irrigation solutions (0.05–0.1%), and wash lotions (0.3%) [106]. OCT solution at the concentration of 0.1% is the most preferred option for root canal treatment because it successfully eliminates *E. faecalis*, *C. albicans*, *S. epidermidis*, and other encountered bacteria in endodontic infections, while also presenting a good tolerability. However, without mechanical instrumentation, disinfection of the root canal would not be possible, because endodontic therapy needs professional cleaning to penetrate the infected areas [107,108,109,110,111,112]. OCT incorporated in mouthwashes at a 0.1% concentration is the main choice for treating gingivitis and periodontitis because it can decrease plaque, bleeding, and the gingival index while also reducing probing pocket depth and clinical attachment loss. Additionally, this formulation is excellent for older people who may not be able to properly brush their teeth because of illness or surgery, or for those with special needs [86,93,99,113,114,115].

OCT is highly effective for wound treatment due to its wide range of pH activity (1.6–12.2 pH) [88,116]. OCT hinders the movement of epidermal Langerhans cells and suppresses the release of several cytokines (IL-8, IL-33, and IL-10) in vitro, indicating its potential anti-inflammatory effects [117]. Due to its anti-inflammatory capacity, it enhances the wound healing process and improves the quality of scars in patients undergoing surgical operations [88]. It is also effective in treating gingival inflammation and reducing dental plaque in patients with fixed orthodontic treatments [90]. OCT facilitates the process of remineralization in demineralized enamel and dentin, which can help go prevent dental caries [89]. The evidence supporting OCT usage is shown in Table 2.

#### 2.2.5. Adverse Effects

Although in some studies, OCT was demonstrated to be more effective than CHX against certain types of bacteria and can be a great alternative for removing dental plaque, it is not without adverse effects [86,121,122,123]. Cationic antiseptics such as octenidine can catalyze browning reactions and cause teeth staining when volunteers consume significant amounts of chromogenic beverages [124]. Other side effects reported in several studies included taste disturbances, burning sensations, a bitter aftertaste, ulcerations, irritation, nausea, and headaches [86]. In one study, subjects who rinsed for 30 s for 5 days with 0.1% OCT mouthwash presented teeth discoloration (*p* = 0.0011), 32 of them reported taste disturbances, 9 tongue discoloration, and 4 headaches [99]. In the worst-case scenario, IgE-mediated anaphylaxis can occur after its use [125]. 

#### 2.2.6. Experimental Methods Used to Evaluate the Efficacy and Safety of OCT in Dental Applications

Different concentrations of OCT and CHG wash-mitts were tested on three fungal strains (two of *C. auris* and one of *C. albicans*) using the quantitative suspension method (EN 13624). The EN 13624 protocol is a test method used for evaluating the fungicidal or yeasticidal activity of chemical disinfectants and antiseptics used in medical settings. The procedure involves mixing interfering substances (bovine albumin solution and erythrocytes), the test suspension, and the sample product, maintaining them for a specified contact time (30 s), neutralizing the mixture, and then after the incubation period, counting the number of colonies. After following the protocol, the results revealed that OCT had fungicidal action at ≥1% against the *C. auris* strains and ≥10% against *C. albicans* after 30 s of exposure. It was also noticed that OCT presented better activity than CHG against the fungal strains [126]. Ugur et al. [22] compared the cytotoxic effects of CHX, OCT, and HA (hyaluronic acid) using the MTT assay and found that OCT had the highest cytotoxic effect on the cells. The results were obtained after adding the MTT solution to the culture for 4 h and then removing it using DMSO to dissolve the formazan crystals. Afterwards, the absorbance was measured at 560–750 λ. For the CHX and OCT groups, the cell viability at 24 h was significantly higher than that at 72 h. The cell viability in both the CHX and OCT applications exhibited a decline at 48 h and then increased at 72 h. Total apoptotic cell differences were assessed by using an Annexin V and dead cell assay. The cells were treated with a staining buffer that contained Annexin V and propidium iodide. Then, the cells were incubated at room temperature and protected from light for 15 min. The cells were then centrifuged, the buffer was aspired, and the cells were suspended in PBS so the samples could be analyzed using a flow cytometer. Cell death was time-dependent and there was a rise in apoptotic levels at 48 h and a subsequent decrease at 72 h for CHX and OCT. According to the wound healing assay (a vertical scratch was traced with a pipette tip through the cells and the closure of the scratched area was measured to quantify their ability to migrate, a critical aspect in tissue regeneration), all three solutions (CHX, OCT, and HA) exhibited an inhibitory effect on HgF migration. The OCT groups showed a significantly worse effect on cell migration. However, another study showed after the MTT assay that 1% OCT mouthrinse had a better effect on gingival fibroblasts and epithelial cells, being less cytotoxic compared to 0.2% CHX at various time points (1, 5, and 15 min), making it a preferable alternative due to its reduced cytotoxicity and fewer side effects. The MTT assay was performed by adding MTT solution to the cells and incubating the plates for 2 h. The solution was then aspirated and DMSO was added to dissolve the crystals. After another 15 min of incubation, the results were examined using a spectrophotometer and an automatic reader. The absorbance was measured at 555 λ for the gingival cells and 550 λ for the epithelial cells [127]. 

Xiaoyu et al. [123] compared the cytotoxic effects of OCT and CHX on human normal chondrocytes, using the MTT assay, and found that chlorhexidine gluconate exhibited a lower toxicity at equivalent doses. The study revealed a dose-dependent decrease in cell viability and proliferation for both compounds. OCT dihydrochloride and CHX were tested at the same concentration of 0.1% and presented cytotoxic effects on human articular cartilage. However, OCT use resulted in a higher toxicity in the cell culture.

#### 2.2.7. Clinical Trials

Researchers tested 0.1% OCT mouthwash in 45 patients with stage I–II periodontitis over a 3-week trial. The patients’ symptoms were evaluated once every week: probing pocket depth was measured by inserting a periodontal probe in the gingival sulcus to record the depth in mm; clinical attachment loss was determined by measuring the distance from the cementoenamel junction to the base of the gingival sulcus; the O’Leary plaque index was evaluated by staining the teeth with a disclosing solution; the Löe and Silness gingival index was determined by visually examining the redness, inflammation, and bleeding of the affected site; oral soft tissue changes were visually examined; and at the end of the trial, a visual analog test was recorded through a self-questionnaire that represented perceptions of the pain and psychological states of the patients during the treatment. After completing these analyses, it was concluded that OCT mouthwash represents a potential alternative for managing stage I–II periodontitis. It improved the gingival index with fewer side effects (teeth staining) than 0.12% CHX mouthwash [93,128,129,130]. A study was conducted on 33 patients with fixed orthodontic treatments to determine if octenidine-based oral antiseptic performs better than standard oral hygiene in improving periodontal status and decreasing periodontopathogens. The trial involved two groups: 22 patients who received OCT-based antiseptic treatment twice daily during the first week of the month, and 11 patients who served as the control group. After 1 month, the probing depth and papillary bleeding index (PBI) were significantly lower in the experimental group compared to the control (*p* < 0.01). The control group showed a reduction in PBI after 3 months. In both groups, the periodontitis-related pathogen levels decreased in subgingival dental plaques during the trial [90]. Jockel-Schneider et al. [99] tested a 0.1% OCT mouthwash in 152 patients to establish its potential to inhibit plaque re-growth and improve gingival health without mechanical plaque control during the trial. The OCT group had a significantly reduced median plaque index and salivary bacterial counts compared to the control at day 5 (*p* < 0.0001). The gingival index also decreased significantly compared to the control (*p* < 0.001). However, discoloration of the teeth was more prevalent in the OCT group (*p* < 0.0011). The symptoms reported in the experimental group were either mild or moderate, with 32 patients experiencing altered taste, 4 having tongue discoloration, and 4 having headaches. These results confirm octenidin’s effectiveness in managing gingival health, with no serious adverse effects.

### 2.3. Povidone-Iodine (PVP-I)

#### 2.3.1. History

**Figure 5:**
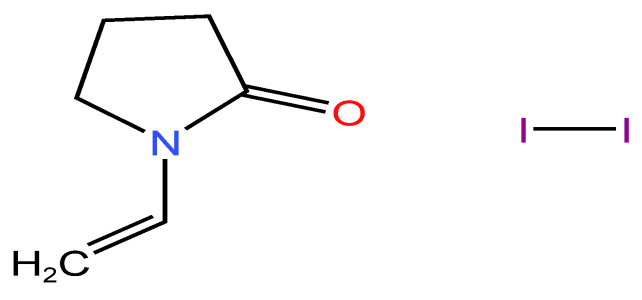
The molecular structure of povidone-iodine.

Elemental iodine, discovered in 1818, revolutionized antiseptic treatments for wound infections, even though it came with certain risks. Povidone-iodine (Figure 5) was not introduced until after 1950, and it represented a safer alternative with a prolonged effect [131,132].

Innovative delivery methods for povidone-iodine in dental applications should be explored, such as slow-release formulations, PVP-I-coated dental materials, and intraoral devices. Additionally, there are a lack of formulations that block the unpleasant taste and odor of povidone-iodine. Creating products that maintain their oxidative power while masking their unpleasant odor and taste is challenging. While a PVP-I composite designed to mask this disagreeable odor and taste was tested in the sinonasal cavity, it has not yet been evaluated in the oral cavity [133,134]. Encapsulating povidone-iodine with edible phospholipids has shown promise in maintaining sustained drug release while combating oral pathogens, though more human studies are needed to assess its efficiency and safety. Clinical studies on PVP-I complexes used in medical device fabrication could potentially prevent infections related to devices such as sutures and drains. This area represents a promising avenue for future research [135,136].

#### 2.3.2. Chemical Structure

Povidone-iodine (PVP-I, iodopovidone, and betadine) is an iodophor composed of molecular iodine and polyvinylpyrrolidone (PVP). Its antibacterial activity stems from the continuous release of free iodine from the hydrophilic complex. PVP acts as a solubilizing carrier, facilitating the steady release of free iodine while maintaining a dynamic equilibrium between the released iodine and the iodine stored within the complex. The continuous release of free iodine is key to its antibacterial activity and hemostatic effect, which is especially important when it is used as a pre-procedural rinse [132,137,138,139].

#### 2.3.3. Antimicrobial Spectrum and Mechanism of Action

Iodine plays a fundamental role in metabolism as a key element in the production of thyroid hormones, and for many years, therapeutic settings have used it as an extremely effective topical antiseptic. Iodine exhibits a wide range of antibacterial properties, effectively targeting bacteria, mycobacteria, fungi, protozoa, and viruses [140,141,142]. It acts against both Gram-negative (*K. pneumoniae, P. aeruginosa,* and *E. coli*) and Gram-positive bacteria (methicillin-resistant *S. aureus*) [134,143]. PVP-I gargles and mouthwashes at 1% (Betadine) are the most frequently used iodinated antiseptics because of the large database available on their safety profiles and bactericidal, fungicidal, and virucidal actions [139]. At 0.5%, it is also beneficial when used as a mouthwash to maintain good oral hygiene. In oral surgery, it is preferred at higher concentrations (10%) to ensure good disinfection [144,145]. It is considered to be generally safe, even at higher concentrations, however, with prolonged use, although rare, it can raise TSH levels, and in the worst cases, induce thyrotoxicosis [146,147]. Nevertheless, one study showed that, after once daily using a mouthrinse with 5% PVP-I, patients presented only a small increase in their TSH levels, but they remained within the normal range [145].

**Figure 6:**
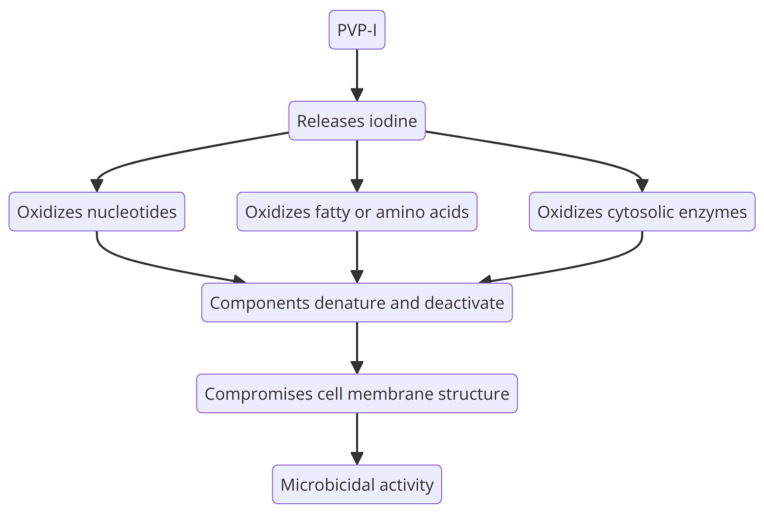
The mechanism of action of povidone-iodine.

Iodine’s microbicidal activity results from its ability to interfere with crucial cellular mechanisms and structures in bacteria (Figure 6). It oxidizes nucleotides and fatty or amino acids in bacterial cell membranes, as well as cytosolic enzymes involved in the respiratory chain. This oxidation process causes these components to denaturate and deactivate. Iodine’s anti-inflammatory effect is related to its ability to collect free reactive oxygen species. PVP-I causes irreparable harm to pathogens because of the released iodine, which interferes with microbial metabolic processes and compromises the structural constituents of the cell membrane [148]. 

#### 2.3.4. Formulations and Current Use of PVP-I in Dentistry

PVP-I is formulated for oral use in mouthwashes, gargles, sprays, and dental flosses in different concentrations ranging from 0.5% to 10%. PVP-I gargles and mouthwashes are typically formulated at a concentration of 1% (*w*/*v*), which contains 0.1 mg/mL of available iodine [133,138,149].

The accumulation of dental plaque contributes to cavity formation. Because povidone-iodine helps to reduce plaque, it can serve as an option for preventing tooth decay [150]. Dental floss coated with PVP-I has been shown to inhibit the production of biofilms and reduce the pathogen levels linked to dental caries development and periodontitis [151]. Another study proved that a concentration of 0.1% can be effective in the treatment of chronic periodontitis when used as a subgingival irrigant [152]. It reduces harmful microbes in the periodontium, improves the attachment of connective tissue in deep periodontal pockets, and reduces periodontal pocket depth when used as a subgingival irrigant or during scaling. The antibacterial effect of a cellulose-based film incorporated with PVP-I showed promising results against pathogens known to be potential risk factors in periodontitis development, such as *C. albicans, P. aeruginosa,* and *Lactobacillus rhamnosus* [153]. The pre-procedural use of 5% PVP-I mouthrinse and subgingival irrigation reduces the risk of bacteremia associated with mandibular third molar extraction and other invasive dental procedures. This practice lowers the presence of pathogens such as α-hemolytic streptococci, β-hemolytic streptococci, and *S. aureus*, which are linked to bacteremia. Although bacteremia is often transient, it can trigger endocarditis in patients with pre-existing cardiovascular diseases. In conclusion, PVP-I is effective not only in reducing the bacterial load associated with bacteremia, but also in decreasing the incidence of other serious complications following surgical interventions [154]. The evidence supporting PVP-I usage is shown in Table 3.

#### 2.3.5. Adverse Effects

Iodine is an indispensable element for the synthesis of thyroid hormones, which plays a crucial function in human metabolism. Iodine deficiency exerts numerous detrimental impacts on growth and development across all age groups. Although high doses of iodine are generally well tolerated, excessive ingestion can impair the functioning of the thyroid gland [158,159].

Povidone-iodine is one of the most commonly used antiseptics available on the market. Although it is generally considered as safe and presents a good acceptability, it can cause some adverse reactions. The Wolf–Chaikoff effect can be triggered by the consumption of excessive quantities of iodine. The excessive use of iodine can also result in iodine-induced hyperthyroidism, which can lead to the development of thyrotoxicosis [147,160,161]. There is a case report of a 50-year-old male patient without thyroid problems who suffered thyrotoxicosis after gargling with a PVP-I solution four times daily for more than 10 years [146]. In contrast, another clinical study conducted on 47 patients who used a 20 mL PVP-I mouthwash twice daily for three weeks showed higher TSH values, but these remained within normal limits [147]. Nevertheless, hypothyroidism can also be a result of long-term iodine use. Significant iodine absorption can cause iodine toxicity in children due to free molecules that can disrupt the blood–brain barrier and cause a variety of systemic symptoms, including convulsions, as shown by a case of seizures induced by PVP-I usage in an 11-year-old [162].

#### 2.3.6. Experimental Methods Used to Evaluate the Efficacy and Safety of PVP-I in Dental Applications

The colony-forming unit (CFU) enumeration method, used before and after treatment application, showed that 5% PVP-I solution efficiently disinfects archwires, brackets, and molars, eliminating *S. mutans* and *L. acidophilus* [163]. 

Another study evaluated the capacity of biofilm formation post-treatment through the irreversible attachment method with 10% PVP-I ointment and 3% PVP-I liposomal hydrogel on *P. aeruginosa*, *C. albicans*, and methicillin-resistant *S. aureus* (MRSA) at different concentrations and time stamps. Both formulations eradicated *P. aeruginosa* after 4 and 24 h at 100% (commercially available concentrations) and 10%. The ointment eliminated the pathogen even at a 33% concentration. Against *C. albicans* and MRSA, PVP-I was effective at a 100% concentration after 4 and 24 h [164].

The in vitro toxicity of povidone-iodine, chlorhexidine, and polyhexamethylene biguanide was analyzed on human fibroblasts (HFs) and keratinocytes (HaCaTs) using the CCK-8 assay and flow cytometry (Annexin V and propidium iodide staining). PVP-I was demonstrated to have the most detrimental effect on both cell cultures. A concentration of 0.5% was highly cytotoxic, even after short-term exposure (5, 30, and 180 s), in a time-dependent manner. Even at the lowest dilution of 1/512, prolonged direct contact resulted in a toxic effect on both HFs and HaCaTs, with the toxicity increasing with the contact time at 0.5, 1, 2, and 4 h. Additionally, 1/512 and 1/64 dilutions altered the cell morphology, causing cytoplasmic shrinkage and exposure of the nuclei. The 1/8 dilution solidified the cells. PVP-I at 1/8 and 1/64 dilutions led to cell apoptosis, because the cells were fragmented to the point of being undetectable [165]. 

The effect of PVP-I on human periodontal ligamental cells was analyzed using an xCELLigence system and the flow cytometry method. A concentration of 0.1 mg/mL did not affect the viability of human periodontal ligamental cells in either ultra-short (10, 20, and 30 s) or short-term (10, 20, and 30 min) periods. However, prolonged exposure to the PVP-I solutions with concentrations of 1 and 2 mg/mL resulted in a significant decrease in cell viability. When exposed to a concentration of 4 mg/mL, the viability of the cells decreased immediately to almost half or lower [166].

#### 2.3.7. Clinical Trials

PVP-I was tested in 60 patients at concentrations of 0.1%, 2%, and 10% as an irrigant for the management of peri-implant mucositis. Gingival health was assessed using the Löe and Silness gingival index (GI) and Mombelli Modified sulcular bleeding index (BI) methods. The solutions were applied after scaling and root planing once a week for four weeks. The best results were observed with the 2% and 10% solutions for both GI and BI [167]. A study was conducted on 16 patients to determine the potential of povidone-iodine to inhibit bacterial growth in the oral cavity of patients on mechanical ventilation. The treatment was applied topically, and the results were examined immediately after at 1 and 3 h. The experimental groups presented a significant decrease in bacterial numbers at all time points. After 3 h, the povidone-iodine group successfully inhibited bacterial growth. This suggests that PVP-I is beneficial in inhibiting bacterial development in oropharyngeal fluid [168]. A comparative study was performed on 105 participants to assess the efficacy of povidone-iodine and hydrocortisone as irrigants during the surgical removal of mandibular third molars. To establish postoperative pain scores and swelling, a visual analog scale was used after 2 and 7 days after surgery. After 2 days, the mean ± SD pain scores were 4.57 ± 0.60 for the PVP-I group, 4.82 ± 0.70 for the hydrocortisone group, and 5.71 ± 0.45 for the control group. After 7 days, the mean ± SD pain scores were 2.82 ± 0.70 for the PVP-I group, 2.62 ± 0.59 for the hydrocortisone group, and 3.34 ± 0.48 for the control group. After 2 days, the mean ± SD swelling measurements were 129.28 ± 6.58 for the PVP-I group, 128.90 ± 5.80 for the hydrocortisone group, and 132.18 ± 8.40 for the control group. After 7 days, the mean ± SD swelling measurements were 125.53 ± 5.79 for the PVP-I group, 125.99 ± 6.57 for the hydrocortisone group, and 126.00 ± 8.66 for the control group. PVP-I presented better results after 2 days in terms of pain scores, however, hydrocortisone was the most effective by the end of the trial. Both the PVP-I and hydrocortisone groups experienced reduced swelling, but there were no significant differences between the groups. These findings suggest that povidone-iodine could be used as an alternative to corticosteroids for managing pain and swelling after surgical extractions [169].

### 2.4. Sodium Hypochlorite (NaOCl)

#### 2.4.1. History

**Figure 7:**
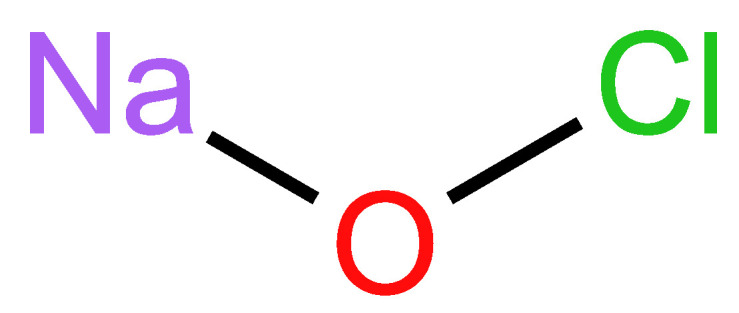
The molecular structure of sodium hypochlorite.

Louis Claude Berthollet, an Italian chemist, initially identified sodium hypochlorite (Figure 7) in 1785. Its primary application was found to be in the process of bleaching (“Javelle water”) [170,171]. The antibacterial activity of sodium hypochlorite was first observed in 1843 when the practice of hand washing between patients resulted in significantly reduced rates of infection transmission, but it was not until 1920 that it was used as an endodontic irrigant [172]. 

Even though sodium hypochlorite is widely used in endodontics for its remarkable antimicrobial properties, there is still an ongoing debate about the optimal concentration for efficacy without compromising safety. More research is needed to establish these concentrations and further explore more advanced delivery systems, such as controlled-release formulations and innovative irrigation techniques, to enhance the drug’s beneficial actions and minimize tissue damage. Current studies suggest that NaOCl’s interactions with the organic material and biofilms within root canals can diminish its effectiveness. As a result, it would be helpful to find ways to improve its biofilm disruption capabilities through combination therapies with other agents or mechanical techniques that could enhance treatment outcomes. Additionally, its long-term effects on dentin structure and mechanical properties remain unclear. It is important to acknowledge how prolonged exposure affects dentin so that new protocols are developed to balance the benefits with the preservation of teeth. Continuous exposure to NaOCl may contribute to bacterial resistance, yet this aspect is not well documented. More research should concentrate on how to avoid this risk of developing resistance, such as by alternating or combining NaOCl with other antimicrobial agents. Exploring the efficacy of sodium hypochlorite in combination with other irrigants or additives could enhance its properties while reducing its adverse effects on dental tissues. Developing and testing new formulations that can reduce unpleasant side effects like taste and odor while maintaining their antimicrobial potency is needed. Clinical trials testing these new formulations are a must to ensure their relevance and safety for dental use [173,174,175,176].

#### 2.4.2. Chemical Structure

Sodium hypochlorite is an inorganic sodium salt that releases active chlorine in water-based solutions. The activity of hypochlorite aqueous solutions depends on the concentration of active chlorine, which results in its disinfectant properties. This activity is inversely proportional to the pH value, making it less effective at higher pH levels. Hypochlorous acid (HClO) is a byproduct of NaOCl when it dissolves in water. HClO’s oxidative capacity makes it an excellent disinfectant, since it oxidizes thiol groups from phagocytized bacteria. This action is crucial in dental applications, such as root canal treatments, gingivitis, periodontitis, and dental caries management. Since almost all oral diseases are associated with bacterial populations, HClO’s ability to disrupt bacterial cells makes it a valuable tool for maintaining oral health and preventing infections [177,178,179,180,181,182]. 

#### 2.4.3. Antimicrobial Spectrum and Mechanism of Action

Sodium hypochlorite is the commonly suggested solution for effectively removing the remaining germs in root canal procedures because of its potent antibacterial and tissue-dissolving properties [183]. It is a broad-spectrum disinfectant that exhibits antibacterial effects on Gram-positive (*Streptococcus* spp., and *E. faecalis*) and Gram-negative (*E. coli* and *Acinetobacter* spp.) bacteria [184,185,186]. Additionally, it presents antiviral and antifungal activity [187,188].

**Figure 8:**
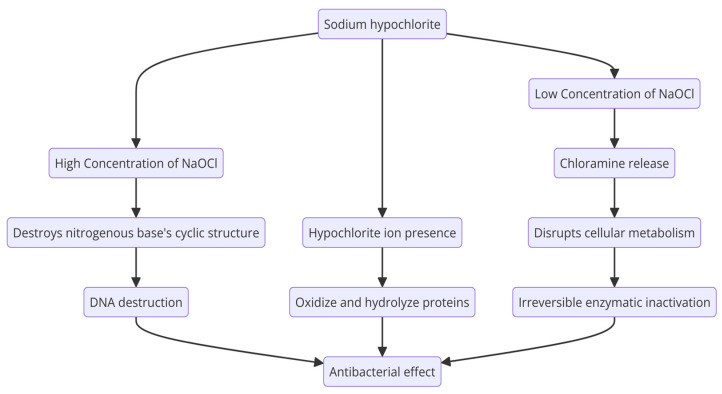
The mechanism of action of sodium hypochlorite.

It oxidizes thiol groups from bacterial enzymes, causes lipid peroxidation, and interferes with cellular metabolism due to chloramine release. The antibacterial activity of sodium hypochlorite is attributed to the hypochlorite ion’s presence and its capacity to oxidize and hydrolyze proteins. The effect of NaOCl on nucleic acids is dose-dependent. At low concentrations of NaOCl, chloramines are formed. Chloramines disrupt cellular metabolism and cause the irreversible enzymatic inactivation of bacteria via oxidative effects. At high doses, it can destroy the nitrogenous base’s cyclic structure and cause DNA destruction (Figure 8) [189,190]. 

NaOCl is most commonly used in endodontic treatments at a concentration of 2.5% due to its good antimicrobial activity and fewer adverse effects. Higher concentrations, such as 5.25%, despite having tissue-dissolving properties, may lead to tissue irritation and increased cytotoxicity [183]. 

#### 2.4.4. Formulations and Current Use of Sodium Hypochlorite in Dentistry

In stomatology, NaOCl comes in gel- or liquid-based formulations [191]. It is frequently utilized at concentrations that vary from 0.5% to 5.25%, with 2.5% being the most commonly used [183].

Dentists primarily use it for postoperative pain management and root canal disinfection due to its beneficial effects in reducing endotoxin levels, strong ability to effectively eliminate biofilms, and capability to dissolve necrotic soft tissue rather than healthy tissue [192,193]. One study showed that 5.25% NaOCl reduces postoperative pain more effectively than a lower concentration (2.5%) [194]. Although it has a limited ability to enter dentinal tubules, a 1% concentration has been shown to efficiently reduce the number of *C. albicans* and *E. faecalis* immediately after a root canal procedure [195]. A comparative study on biofilm removal demonstrated that NaOCl exhibited the strongest antibacterial effect among PVP-I, CHX, curcumin, and Triphala [155]. Additionally, it possesses antimicrobial properties against the microorganisms found in dental plaque, leading to a reduction in gingivitis [180,196]. The evidence supporting NaOCl usage is presented in Table 4.

#### 2.4.5. Adverse Effects

NaOCl requires a minimum contact time with the tissue to effectively exert its activity. At higher doses, it can negatively impact the viability of cells and induce apoptosis and tissue irritation [183]. A predominant adverse impact that is commonly mentioned is the disagreeable taste of bleach. Additionally, there have been reports of tooth staining, tongue irritation, and a burning sensation [198]. In another study conducted on 23 patients using a 0.2% NaOCl solution, it was confirmed that the most prevalent side effect for most participants was the unpleasant taste of bleach. Others complained about tooth staining, dry mouth, taste alterations, and mucosal irritation. However, long-term negative effects were unlikely to persist or occur [199].

#### 2.4.6. Experimental Methods Used to Evaluate the Efficacy and Safety of NaOCl in Dental Applications

It was observed through the qPCR method that NaOCl had a dose-dependent negative effect on the viability, proliferation, and differentiation of dental stem cells [200]. In a study conducted by Sawada et al. [201], the cytotoxic effect of 0.25% NaOCl on bone viability was reported to be the highest of all the other substance groups (CHX, CHG, PVP-I, and hydrogen peroxide) at all time stamps (1, 5, 10, 20, 30, and 60 min). In a recent study, the negative effects of 0.08% NaOCl on osteoblasts were analyzed. It was concluded that NaOCl exhibited toxic effects on the cells even after brief exposure periods of 2, 5, and 10 min. The study employed an XTT assay to quantify cell viability, fluorescence microscopy to distinguish living cells (stained with fluorescein diacetate) from dead cells (stained with propidium iodide), and lactate dehydrogenase activity to indicate cell necrosis by marking the loss of plasma membrane integrity [202]. Prior research has already shown through MTT assay, ELISA kit, and SRB assay that NaOCl has harmful effects in a dose-dependent manner on human gingival fibroblasts. A concentration of 2.5% significantly reduced cell viability and a concentration of 5.25% caused DNA damage through oxidative processes [203,204]. 

Different NaOCl irrigation systems (2.5% NaOCl+conventional needle and 2.5% NaOCl+EndoVac) were tested in vivo on deciduous molars. Both pre- and postoperative samples were placed in the canal for approximately 1 min and sent for microbiological testing. The samples were added to a special broth designed to support bacterial growth, then were vortexed, diluted, transferred to blood agar media, and incubated for 24–48 h. CFUs were used to estimate the numbers of viable bacteria by counting the colonies formed on the agar plates. It was revealed that 2.5% NaOCl+EndoVac was the most effective out of the two root canal irrigation systems in decreasing the bacterial load in deciduous teeth [205].

#### 2.4.7. Clinical Trials

Another study proved sodium hypochlorite’s beneficial action as a root canal disinfectant. NaOCl gel and NaOCl aqueous solution were tested at 5% concentrations on 42 subjects with multirooted teeth with pulpal necrosis. A pre-treatment sample was obtained from the largest canal, and the two other samples were acquired after 60 s of exposure to NaOCl formulations. A microbiological assay was performed to calculate the bacterial load after the sample was placed on a brain–heart infusion agar medium and incubated for 48 h. Both formulations reduced pathogen levels with comparable efficacy, although the aqueous solution presented stronger antimicrobial action [206]. A study was conducted on 298 patients to analyze the efficiency of two different concentrations of NaOCl in root canal treatment. The subjects were divided into two groups: Group 1 (n = 153) who received 0.5% NaOCl and Group 2 (n = 145) who received 3% NaOCl. After irrigation with the solution, samples were collected, and the patients were examined 7 days post-surgery. The best results were obtained from the 0.5% group, with only 13.4% positive cultures, compared to the other 3% NaOCl group with 18.6%. Additionally, less swelling was observed in Group 1 (5.1%) than in Group 2 (17.8%). No significant differences were found between these concentrations in terms of efficacy. These results suggest that a lower concentration of NaOCl might be preferred for root canal treatment, as it can minimize the risk of adverse effects while also being effective [207].

### 2.5. Cetylpyridinium Chloride (CPC)

#### 2.5.1. History

**Figure 9:**
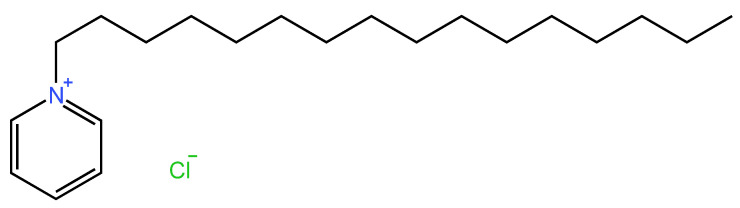
Molecular structure of cetylpyridinium chloride.

Cetylpyridinium chloride (Figure 9) is a quaternary ammonium compound (QAC) that is widely used in several dental treatments. Since their employment in 1930, QACs have been significant health tools due to their antiseptic properties on the skin and mucous membranes. However, CPC’s antimicrobial activity was first described in 1939 [208,209].

The use of CPC for dental applications remains largely unexplored in the realm of newer delivery systems. Such innovations would help to sustain its antimicrobial action. Moreover, these innovations have the potential to enhance its penetration into dental tubes, which is particularly crucial in endodontic treatments. Moreover, there is currently insufficient evidence regarding the safety of its prolonged use. Because of this, more research should focus on chronic exposure and outcomes to ensure that CPC use is safe for patients even after longer periods [28,210,211,212,213].

#### 2.5.2. Chemical Structure

Cetylpyridinium chloride is an amphiphilic cationic compound with a central nitrogen atom connected to at least one hydrophobic side chain [208]. Its antimicrobial activity is linked to its amphiphilic nature through various mechanisms of action. The cationic part is responsible for its anti-plaque effect due to the attachment it creates with the negatively charged bacterial wall, which allows it to kill bacteria. For this reason, its molecular structure is closely related to its beneficial effect on plaque-associated diseases [213,214,215]. The molecule’s lipophilicity is determined by the presence and length of its alkyl chain [216]. 

#### 2.5.3. Antimicrobial Spectrum and Mechanism of Action

It has a broad spectrum of action against Gram-positive and Gram-negative bacteria, fungi, and viruses [217,218]. Despite discussions about its effect on Gram-negative bacteria, one study proved otherwise [219]. 

**Figure 10:**
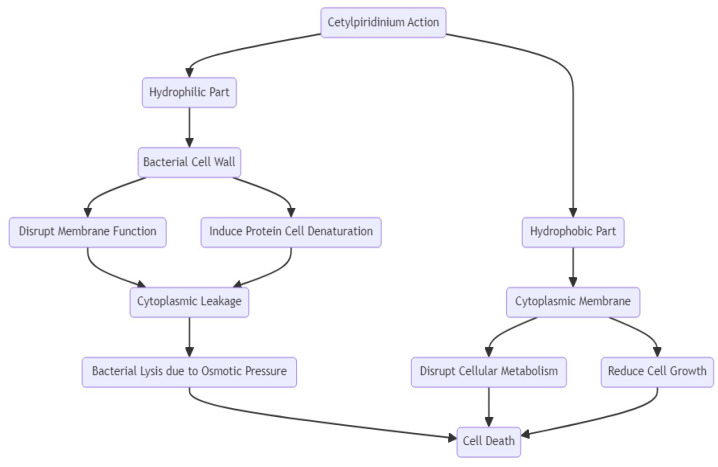
The mechanism of action of cetylpiridinium chloride.

It is believed that the interaction of CPC with bacteria occurs through the disruption of membrane function or the induction of protein cell denaturation, resulting in cytoplasmic leakage and bacterial lysis due to osmotic pressure (Figure 10). This process occurs because of the interaction between the positively charged sites of CPC and the negatively charged bacterial cell wall. Its amphiphilic character directly impacts its antibacterial activity. Its hydrophobic component is responsible for membrane damage as it penetrates the cytoplasmic membrane, disrupting the cellular metabolism, reducing cell growth, and inducing cell apoptosis. The cationic hydrophilic part of the CPC molecule is crucial for its antimicrobial activity. It binds strongly to negatively charged bacterial cells, leading to the disruption of membrane integrity and ultimately resulting in cell death [215,216]. Cetylpyridinium chloride is commonly used in oral care products at concentrations of 0.05%, 0.07%, and 0.1%. Researchers have reported adverse reactions at all these doses. In one study, at a concentration of 0.05%, CPC caused teeth sensitivity, glossodynia, dysgeusia, and oral discomfort. Another trial also reported taste alteration, tongue numbness, and a burning sensation after using 0.05% CPC mouthwash. One study found that using a concentration of 0.07% resulted in tongue numbness, taste alteration, tooth staining, gingival bleeding, and subgingival swelling. At a concentration of 0.1%, CPC caused non-serious adverse reactions in a clinical trial. According to the Scientific Committee on Consumer Safety of the European Union, mouthwashes containing CPC at 0.1% (w/w) are the maximum limit for gingivitis treatment. Additional clinical studies are required to evaluate the correlation between dosage, efficacy, and safety across a wider range of dental applications [213,220,221].

#### 2.5.4. Formulations and Current Use of Cetylpyridinium Chloride in Dentistry

It comes in several formulations, such as mouthrinses, toothpastes, lozenges, mouth sprays, throat sprays, nasal sprays, and chewing gum [222,223,224,225,226].

CPC is used in stomatology as an anti-plaque and antimicrobial agent. Plaque accumulation is a known factor that can contribute to gingival inflammation and periodontal disease. The antiplaque effect is correlated with its cationic component, which attaches to the negatively charged bacterial wall, prevents bacterial growth, and induces cell death via osmotic pressure and bacteriolysis, causing the cellular components to leak [214]. CPC’s antibacterial action makes it a great option for dental health management. It helps to reduce *Streptococcus* spp., which primarily causes tooth decay, one of the most common dental problems [227,228]. Evidence supports its potential beneficial role in endodontic treatment. It presents synergistic activity with silver ions against *E. faecalis* growth, which is a known “stubborn” bacterium often encountered in root canal and periradicular infections. However, more studies are required, in this sense, to prove its safety when used as a root canal irrigator [229,230,231]. Throughout the COVID-19 pandemic, CPC proved to be an effective strategy for reducing the viral load in the oral cavity and limiting the transmission of SARS-CoV-2 during dental procedures. Its mechanism of action is hypothesized to occur through viral envelope disruption [232]. The evidence supporting CPC usage is shown in Table 5.

#### 2.5.5. Adverse Effects

Some patients complained about a burning sensation in the mouth after CPC usage. It has also been reported to cause irritation, ulceration, teeth discoloration, and exfoliation of the oral mucosal epithelium, as well as a burning sensation [235]. In a study conducted on 25 subjects who used a 0.05% CPC and hyaluronic acid mouthrinse twice daily, five of the patients reported that mouth ulcerations were the most encountered unpleasant outcome [236]. Other studies reported adverse reactions in a total of 53 patients. Among them, 15 experienced teeth staining, tongue numbness, taste disturbances, gingival bleeding, and subgingival swelling. Additionally, 25 patients presented with teeth and tongue staining (which disappeared after the trial finished), 2 reported tooth sensitivity, 2 had mouth ulcers, 3 experienced glossodynia, 2 reported dysgeusia, and 1 experienced oral discomfort [213,237].

#### 2.5.6. Experimental Methods Used to Evaluate the Efficacy and Safety of CPC in Dental Applications

The combination of 50 µM CPC and 500 µM bismuth lipophilic nanoparticles was tested against *E. faecalis.* Antibacterial activity was detected after the disk diffusion assay by measuring the inhibition zone using a Vernier caliper. The measurements revealed a larger zone of inhibition after the treatment application, indicating a bactericidal effect against the pathogen. After performing the live/dead assay and fluorescence microscopy, the antibiofilm assay could be determined. It was observed that the CPC nanoparticles decreased the bacterial biofilms almost completely after 24 h. These results suggest CPC’s beneficial action in reducing the incidence of surgical site infections [238].

In one study, CPC’s toxic effects on human gingival epithelial progenitor cells were tested using a real-time cell analysis and Annexin-V. Its maximum toxicity on the cells was observed in less than 24 h at a half-minimal inhibitory concentration of 0.0003% [239].

Another study investigated the cytotoxic effects of some OTC mouthrinses that contained cetylpyridinium chloride on human gingival fibroblast cells, HSC-2s (human oral epithelial carcinoma cells), and L929s (murine aneuploid fibrosarcoma cells). The results were obtained after the formazan formation assay and visual inspection after the cells were stained with trypan blue. Cell viability was then examined under a microscope. The CPC mouthrinses had an LD_50_ of 0.06%, demonstrating a highly cytotoxic effect on the cell cultures [240]. Two commercially available mouthwashes with concentrations of 0.045% and 0.075% CPC had their biocompatibility tested on L929 mouse fibroblasts. The MTT assay and a real-time cell analysis were performed to examine the viability and proliferation rate of the cell line, and phase-contrast microscopy was used to evaluate morphological changes. A reduction in cell viability and their ability to proliferate was observed. Also, cell morphology was affected after treatment with a 1:1 dilution and 2 min of exposure. The cells appeared rounder and detached, a sign of toxicity [241].

#### 2.5.7. Clinical Trials

The effect of 0.045% CPC mouthrinses was tested on 40 subjects by measuring oral health parameters such as the tongue coating index, gingival index, plaque index, and the presence of some volatile sulfur compounds. Aerobic bacteria levels were also measured after the samples were plated on blood agar and the colonies were counted after 16–18 h of incubation. The results confirmed that the CPC mouthrinses had antimicrobial and anti-halitosis effects, indicating their potential for treating gingivitis and other oral problems associated with plaque accumulation [242]. A 0.05% CPC mouthwash was compared to a 7% PVP-I gargle to establish their roles in perioperative patient care. The patients received treatment before surgery, and they were examined after one week. Both products significantly reduced gingival bleeding (*p* < 0.001). However, only the CPC mouthwash suppressed Streptococcus levels after one week of surgery [243].

### 2.6. Hydrogen Peroxide (HP)

#### 2.6.1. History

**Figure 11:**
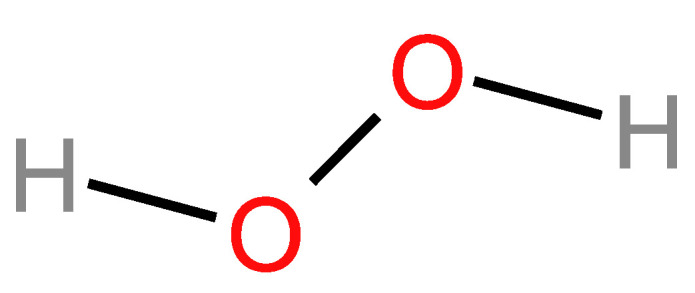
Molecular structure of hydrogen peroxide.

Hydrogen peroxide (Figure 11) has been used as a bleaching agent and disinfectant since its discovery in 1818. [244]. However, until the end of the 19th century, it had not been obtained in its pure form [245]. It was first employed in dentistry in 1913, when it was used to control “pyorrhea”, also known as periodontitis, by reducing plaque accumulation [246].

Despite its extensive use, there are several potential areas for further study to enhance its efficacy, safety, and application techniques. Hybrid irradiation with hydrogen peroxide was demonstrated to enhance the potency of bleaching while lowering the incidence, severity, and duration of dentin hypersensitivity through intrinsic biostimulation. These results must be further confirmed in human trials to ensure that it is truly capable of improving patients’ overall experience and comfort. Further studies are necessary to analyze the release kinetics of H_2_O_2_ from whitening products to observe the long-term effects on tooth structure and safety after longer periods of use. Additionally, the correlation between the release kinetics and the bleaching effect should be determined in other studies. More trials should be conducted to determine the antimicrobial efficacy of H_2_O_2_ with and without photoactivation on various pathogens, from dental implants to combating peri-implantitis. Additionally, the therapy for this condition could be expanded by also investigating the mechanism of action of photoactivation on a wider spectrum of microorganisms. Hydrogen peroxide fumigation in dental offices is another area that is worth exploring due to the high importance of maintaining a sterile environment in dental practice [247,248,249,250].

#### 2.6.2. Chemical Structure

Hydrogen peroxide (H_2_O_2_) is the simplest peroxide. It is an unstable oxidizing agent that can cause combustion when it comes into contact with organic material [251,252]. The release of oxygen molecules is what makes it an excellent teeth-whitening agent, and also helps when it comes to treating halitosis, gingivitis, and periodontitis [253,254,255,256].

#### 2.6.3. Antimicrobial Spectrum and Mechanism of Action

Hydrogen peroxide is used as an antiseptic. It is effective against Gram-positive and Gram-negative bacteria, as well as bacterial spores. It also possesses virucidal and fungicidal properties [257].

**Figure 12:**
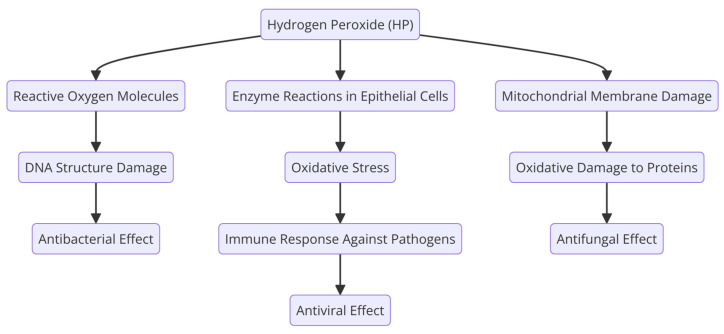
The mechanism of action of hydrogen peroxide.

HP degradation is responsible for its antibacterial effect (Figure 12). After the degradation process starts, reactive oxygen molecules are released and they interfere with the DNA structure, causing it to break due to oxidation [257]. Its mechanism of action against viruses is quite similar. HP induces enzyme reactions in the epithelial cells of the oral mucosa, causing oxidative stress that triggers an immune response against pathogens [258]. On fungi, it acts by disrupting the mitochondrial membrane by causing oxidative damage to proteins [259].

Hydrogen peroxide has a dose-dependent effect. In one study, even at a concentration of 0.8%, it caused a burning sensation for two patients, pinching for two patients, and both for one patient. A concentration of 3% caused no tooth sensitivity, but 12 patients had oral lesions after the treatment. In a meta-analysis of 25 studies, it was shown that there is no need to use higher concentrations of H_2_O_2_ (35–40%) to achieve the best results, as lower (6–15%) and medium (20–30%) concentrations can result in the same bleaching effect with less adverse effects. It was shown that there is a 33% lower chance of experiencing side effects when using lower or medium concentrations instead of higher ones [253,254,260,261].

#### 2.6.4. Formulations and Current Uses of Hydrogen Peroxide in Dentistry

It can be found in mouthwashes, toothpastes, and gels. Its concentrations vary based on the purpose. In OTC products such as mouthwashes, these concentrations range from 1.5 to 3%. The products available on the market can help with plaque reduction, dental caries, and halitosis. However, in Europe, products that are above 0.1% are not allowed to be sold to the general public. Teeth-whitening gels are available at concentrations ranging from 30 to 35% for in-office use (sometimes up to 40%) [262,263,264,265,266].

Since 1982, people have used hydrogen peroxide as a teeth-whitening agent. The release of free radicals from the HP in the dentin is believed to be the mechanism underlying this whitening effect. After the free radicals interact with the dentin, they break down the double bonds of the organic pigments, creating the effect of whiter teeth due to structural changes [255]. It is also used as an effective treatment for gingivitis, reducing the plaque index even at concentrations as low as 0.8% [254]. Since making its name in the dentistry field as a treatment for periodontitis, it is still used for this purpose today. Today, newer approaches like diode lasers are used to better deliver hydrogen peroxide to the affected areas in periodontal disease [267,268]. The evidence supporting HP usage is shown in Table 6.

#### 2.6.5. Adverse Effects

Because it is a strong oxidizing agent, it should be used carefully at home to prevent mouth infections, gum burns, and possible damage to the enamel and teeth nerves [270].

When it is used as a teeth-whitening agent at high concentrations, it can have mutagenic and genotoxic consequences because it induces oxidative stress. Its overuse can also cause gingival irritation and tooth demineralization. However, tooth sensitivity is the most common adverse reaction [271,272]. This claim is supported by a clinical trial performed on 92 patients who bleached their teeth with 4% HP for 30 and 120 min, and both groups presented tooth sensitivity. Despite the high number of participants who suffered tooth sensitivity (32 from the 30 min group and 37 from the 120 min group), the pain and discomfort had low intensity [273]. In a study conducted on 38 patients (divided into two equal groups) who bleached their teeth with 6 and 15% HP, tooth sensitivity (29.6% from the 6% HP group and 44.4% from the 15% HP group) and gingival irritation (57.7% after 6% HP treatment and 53.8% after 15% HP treatment) were reported [274]. Low concentrations (up to 9%) cause transient symptoms such as blisters [275].

#### 2.6.6. Experimental Methods Used to Evaluate the Efficacy and Safety of HP in Dental Applications

In one study, the MTT assay revealed after 4 h that 3% H_2_O_2_ mouthwash had a cytotoxic effect on fibroblasts. Using an inverted phase-contrast microscope, it was observed that HP altered cell morphology by causing shrinkage, membrane blebbing, and condensed nuclei [276]. Gutiérrez-Venegas et al. [277] showed by using Hoescht 33258 staining and a TUNNEL assay that H_2_O_2_ at 100 µM induced cytotoxic effects on human gingival fibroblasts at all time stamps (30, 60, and 90 min). Another indicator of cell death was demonstrated after observing the activation of Caspase-9 (in a time-dependent manner), Caspase-3 (after 30 min of exposure), a decrease in Bax expression (time-dependent), and an increase in Bcl expression (time-dependent) using Western blot analysis. According to another study, it was reported that, after performing a resazurin-based assay using fluorometric methods, H_2_O_2_ solutions at concentrations ranging from 0.05 to 10 µg/mL exhibited cytotoxic effects on human fetal osteoblasts and human gingival fibroblasts. Even at the lowest concentration of 0.05 µg/mL (the reported safe concentration is 0.68 µg/mL), it decreased HGF cell viability by 50%. It was observed that it induced moderate to severe cytotoxic effects in both cell cultures, reducing cell viability by around 50% in osteoblasts and to under 50% in HGF cells after 1 h of exposure. After examination with an inverted phase-contrast microscope, changes in cell morphology were also observed, with a decrease in cell density and detached cells [278].

#### 2.6.7. Clinical Trials

In another study, 6.5% hydrogen peroxide whitening strips were tested on 30 subjects to evaluate their efficacy against tetracycline stains in a 6-month trial. The results were evaluated using the Vita shade guide to assess the changes in color from the baseline. Half of the patients experienced moderate adverse effects, such as temporary mild tooth sensitivity and oral discomfort, during the first three months following daily 1 h (two sessions of 30 min each) application. After the 6-month trial, an improvement in tooth color was noticed, with a good tolerability during treatment [279]. Another study investigated HP effectiveness and tooth sensitivity following in-office bleaching on 40 participants at 15% and 35% concentrations. The participants were divided into two groups of 20 (Group A and Group B). Group A administered HP 15% for 144 min over three equal sessions of 48 min and Group B administered HP 35% for 135 min over three equal sessions of 45 min. A visual scale analog and reflectance spectroscopy were used to examine the effect of hydrogen peroxide on tooth sensitivity and its effectiveness in whitening the teeth. After interpreting the results, it was found that the lower HP content bleached better and caused reduced tooth sensitivity [280].

### 2.7. Safety Profiles of Synthetic Compounds

#### 2.7.1. Chlorhexidine

Chlorhexidine remains at the top of the list in dentistry due to its many benefits achieved in a short time. Despite its numerous positive actions, chlorhexidine should be used cautiously and for a limited duration due to its adverse effects. CHX 0.12% mouthwash is an excellent plaque-removing agent that helps in the treatment of gingivitis, however, in a clinical trial, it caused extrinsic tooth staining in many patients [68]. Another study confirmed the benefits of CHX mouthwash in treating gingivitis at a concentration of 0.12%, but once again, patients complained about side effects, including tongue numbness, mild irritation, parotid gland discomfort, and parotitis, and two patients dropped out because of the gingival desquamation and burning sensations [69]. Mihajlo et al. [70] conducted an online survey with 41 dentists to determine whether they suggested that patients use 0.12% CHX mouthwash as an adjunct to oral hygiene maintenance. The majority of them would recommend its use for halitosis, gingivitis, and periodontitis, for patients with orthodontic appliances, and for other patients with special needs who cannot maintain quality oral hygiene, although adverse reactions like taste disturbance, xerostomia, and tooth discoloration occurred. Chlorhexidine’s severe allergic reactions in the dentistry field are quite rare, but they should not be overlooked, as they can be deadly. In one case, a patient suffered anaphylaxis after using a 1% CHX gel, one woman due to using a 0.2% CHX mouthwash, and three other children because of antiseptic tablets. Another patient had urticaria and skin lesions after using a 0.05% CHX mouthwash [21,71,72,74,75].

#### 2.7.2. Octenidine

Octenidine is most commonly used for removing dental plaque. It has benefits for treating gingivitis and periodontitis. Octenidine has gained more attention because it can substitute chlorhexidine for various problems due to its fewer adverse effects. For instance, studies have demonstrated that endodontic treatment can use a higher concentration of octenidine (≥0.1%) as an intracanal irrigant during chemomechanical debridement, rather than 2% CHX. Another benefit comes from its resilience to the presence of blood, mucin, and albumin, unlike chlorhexidine [121]. However, the main reason octenidine is sometimes preferred over CHX is due to the lower incidence of unpleasant outcomes. Studies have reported that OCT mouthwashes can cause teeth and tongue staining, but in one study, those were mild. Other side effects encountered when using 0.1% and 0.2% mouthwashes for reducing plaque and gingivitis were taste disturbances, burning sensations, ulcerations, irritation, nausea, and headaches. These adverse effects were reported to be dose-dependent [86,99]. It can cause contact dermatitis and IgE-mediated reactions, however, there are reports of anaphylaxis in only two studies’ abstracts [125].

#### 2.7.3. Povidone-Iodine

Povidone-iodine is mainly used as a presurgical disinfectant. It is considered to be generally safe, even after a longer period of usage. However, there was a case report of a toxic reaction after prolonged use (>10 years) [146]. Another study reported that, after using PVP-I for 3 weeks for the treatment of pharyngitis and tonsillitis, it raised TSH levels, however, this was nothing serious, as they were still within normal limits [147]. An 11-year-old who was administered a PVP-I-impregnated antra pack for the treatment of suppurative osteomyelitis of the right maxilla suffered from seizures 60 h post-surgery. Although it is considered to be safe, it has been shown, in some cases, that the quantity of the PVP-I that is absorbed can cause toxic adverse effects, especially in vulnerable people, such as children [162].

#### 2.7.4. Sodium Hypochlorite

NaOCl is used in gingivitis, periodontitis, and mostly as a root canal disinfectant. In some studies, NaOCl at 0.05% and 0.25% decreased dental plaque, supragingival biofilm, gingival inflammation, and bleeding. However, in three of these studies, all participants reported an unpleasant taste of bleach, and in one, 35% reported this effect. In another one, all patients reported extrinsic tooth stains, redness of the tongue (35%), and burning sensations (45%) [198]. It was demonstrated to be effective as an adjunct treatment for patients with stage III–IV periodontitis, as it improved the clinical parameters in patients with severe periodontitis, such as probing pocket depth (*p* = 0.0005) and bleeding on probing (*p* < 0.0001) after 3 months. Additionally, the median percentage of undetectable levels of bacteria in the test group was 64.0%, while in the control group, it was 22.0%. During the treatment, patients complained about the unpleasant taste of bleach, tooth staining, dry mouth, taste alterations, and mucosal irritation. However, long-term negative effects were unlikely to persist or occur [199].

#### 2.7.5. Cetylpiridinium Chloride

CPC is generally considered to be safe and does not cause serious adverse reactions. In one study, CPC was found to be effective at reducing plaque buildup and preventing gingivitis. It was generally well tolerated, although 5 out of 25 patients did report mouth ulcerations after use [236]. Despite these side effects, other studies have confirmed the benefits of CPC in treating gingivitis. For example, 15 patients using a 0.07% CPC solution experienced side effects like teeth staining, tongue numbness, taste disturbances, gingival bleeding, and sublingual swelling. Conversely, only three subjects using a combination of CPC and 0.28% zinc lactate reported adverse reactions, such as a bitter taste and dentin hypersensitivity. In additional studies, 25 patients reported no serious adverse effects, with any teeth and tongue staining being temporary. Other side effects included tooth sensitivity, mouth ulcerations (two cases), glossodynia (three cases), dysgeusia (two cases), and oral discomfort (one case) [213,237].

#### 2.7.6. Hydrogen Peroxide

Hydrogen peroxide is an excellent teeth-whitening agent that has proven its beneficial role in enhancing people’s appearance. It is a popular bleaching product because of its strong oxidizing effects. It has been demonstrated that its effect is not diminished by concentrations of 20%or lower. In fact, these lower concentrations are preferred because they result in fewer adverse effects. Although generally considered as safe, temporary tooth sensitivity is commonly reported even at concentrations of 4%, 6%, and 15% [273,274,275].

In Table 7, the adverse of the synthetic drugs discussed throughout this paper are presented, along with the differences in their intensity and frequency.

## 3. Conclusions

This review highlights the importance of synthetic compounds for both preventive and curative treatments in dental care. It offers an overview of synthetic compounds, most importantly, of their complex and diverse natures in terms of mechanisms of action, efficacy, and safety. Synthetic compounds remain the preferred option for in-office dental use due to their extensive research base and more targeted and faster therapeutic effects. However, despite their versatility and specific mechanisms of action, they come with a high rate of adverse effects, which can be especially unpleasant for vulnerable populations, such as children, older people, and patients with debilitating diseases. By using certain methods and assays, the efficiency and safety profiles of therapeutic compounds can be evaluated. Also, we can gain a broader perspective and a better understanding of their mechanisms of action, allowing for the development of newer and safer products with a higher bioavailability and facilitated effects.

## Figures and Tables

**Table 1:** Supporting evidence for CHX usage.

Uses	Formulations	Type of Study	Spectrum	Results	Ref.
Dental caries	CHX 35% varnishes, CHX 1% gel	In vitro	*Lactobacillus rhamnosus*	Bacterial numbers showed little to no change on the 40 bovine root dentin samples (*p* > 0.05). It is unlikely to prevent root caries from gaining prevalence.	[45]
Dental caries	CHX 0.2% mouthrinse	Controlled trial	*Streptococcus mutans*	It significantly ↓ the amount of *S. mutans* in saliva after using 2.5 mL of the mouthwash for 1 min, twice a day. It may be beneficial for dental caries prevention in children. However, the herbal mouthwash presented better results with fewer side effects at the end of the 15-day trial (*p* = 0.002).	[57]
Gingivitis	0.12%, 0.2% CHX mouthwashes	Systematic review	-	It effectively ↓ dental plaque formation, which can further lead to gingival inflammation.	[58]
Gingivitis	1% CHX toothpaste	Home usage trial	-	The prevalence of gingivitis was significantly ↓ by the end of the 6-month trial compared to the control (*p* < 0.001)	[59]
Periodontal disease	CHX chip (2.5 mg)	Randomized clinical trial	-	It ↓ the probing depth and plaque index. The CHX chip could be an adjunctive therapy for scaling and root planing in periodontitis.	[32]
Root canal treatment	Chitosan CHX-nanoparticles (CS-CHX NPs), 2% CHX gel	In vitro	*Enterococcus faecalis, Candida albicans*	Both formulations significantly ↓ the pathogens. In a shorter period, the CS-CHX NPs formulation was comparable to the gel one, but the antimicrobial activity was significantly higher compared to the other medicament groups (*p* < 0.001). They proved to be beneficial in decreasing the most commonly encountered pathogens in root canal infections.	[14]
Peri-implant mucositis	0.12% CHX mouthwash	Randomized controlled trial	-	It ↓ plaque formation, bleeding on probing, and probing depth (*p* = 0.01). It was demonstrated to be a useful adjunct treatment for mechanical debridement for peri-implant mucositis.	[16]
Biofilm formation	0.5% *w*/*w* CHX spray	In vitro and in vivo	*Staphylococcus aureus*	The inhibition of *S. aureus* was observed in vitro and antiseptic activity was observed in vivo (*p* < 0.05). This confirms that CHX spray can decrease biofilm formation, thereby reducing the incidence of other dental problems.	[60]

Abbreviations: ↓ = reduced; min = minute; mL = milliliter; mg = milligram; *p* < 0.05 = significant differences; *p* > 0.05 = no significant differences.

**Table 2:** Supporting evidence for OCT usage.

Uses	Formulation	Type of Study	Spectrum	Results	Ref.
Biofilm formation	0.1% OCT mouthwash	Crossover study	-	The biofilm thickness appears to be significantly ↓ on the 60 enamel samples when the mouthwash is used once every 12 h for 30 s, making OCT a great candidate for combating several dental problems. The biofilm thickness was 12.32 ± 6.58 µm in control compared to 0.54 ± 0.09 µm after 48 h post-treatment.	[118]
Dental caries	1%, 1.5% OCT added to a resin composite	In vitro	*Streptococcus mutans*	The composite resin increased OCT’s antibacterial action at both concentrations against *S. mutans* after 30 days (*p* < 0.05), demonstrates its beneficial role in caries prevention.	[119]
Gingivitis, periodontitis in patients following orthodontic treatment	1% OCT mouthwash	Systematic review	*Streptococcus mutans*, *Lactobacilus* spp.	It ↓ microbial growth, helping with plaque-induced gingivitis and periodontal damage. It also prevents white spot lesions in patients following orthodontic treatment.	[86]
Root canal treatment	0.0125%, 0.025%, 0.05%, 0.1% OCT solution	In vitro	*Staphylococcus epidermidis*	OCT significantly ↓ the pathogen levels at all concentrations in a dose-dependent manner compared to control (*p* < 0.05). Notably, at the concentration of 0.1%, it exhibited the most potent antibacterial activity, completely eradicating *S. epidermidis*.	[110]
Yeast infection	0.001%, 0.05%, 0.1% OCT mouthwashes	In vitro	*Candida albicans*, *Candida glabrata*	The 0.001% concentration was not sufficiently effective against the strains. OCT at 0.05% and 0.1% successfully inhibited *C. albicans*, even after 30 s of exposure. Only the 0.1% concentration was yeasticidal against *C. glabrata* within 2 min of contact time.	[120]

Abbreviations: ↓ = reduced; s = seconds; h = hours; min = minutes; *p* < 0.05 = significant differences.

**Table 3:** Supporting evidence for PVP-I usage.

Uses	Formulation	Type of Study	Spectrum	Results	Ref.
Biofilm reduction	10% PVP-I solution	In vitro	α-hemolytic *Streptoccocus* sp., *Hafnia alvei*, *Bacteroides ureolyticus*, *Actinomyces odontolyticus*, *Actinomyces meyeri*, *Finegoldia magna*, *Parvimonas micra*	Ten samples were collected from patients with pulpitis who had necrotic pulp. The samples were divided into two groups (A and B). In sample A, α-hemolytic *Streptococcus* sp., *H. alvei*, *B. ureolyticus*, *A. odontolyticus*, and *A. meyeri* were present, and in sample B, α-hemolytic *Streptococcus sp.* strains, *F. magna*, *A. meyeri*, and *P. micra* were present. After 15 min, both samples had a significantly ↓ CFU count (sample A—*p* = 0.000152; sample B—*p* = 0.000311). In both samples, pathogen levels were fully eliminated after 40 min. These results support PVP-I’s beneficial action in endodontics.	[155]
Biofilm reduction	1%, 7.5% gargle, 0.45% throat spray	In vitro	MRSA, *Escherichia coli*, *Enterococcus faecium*, *Streptococcus pyogenes*, *Streprococcus mutans*, *Streptococcus sanguinis*, *Streptococcus pneumoniae*, *Streptococcus agalactiae*, *Pseudomonas aeruginosa*, *Haemophilus influenzae*, *Klebsiella pneumoniae*, *Staphylococcus epidermidis*, *Candida albicans*, *Candida glabrata*, *Coxsackievirus A16*, *Enterovirus 71*	It showed antibacterial (>5 lg reduction) and antifungal (>4 lg reduction) activity after 30–60 s of exposure, as well as antiviral (>4 lg reduction) effects after 0.5–30 min at all concentrations against all pathogens.	[156]
Dental caries	10% PVP-I solution	Controlled trial	*Streptococcus mutans*	The study included 25 children who were divided into 2 groups: Group 1–13 experimental children and Group 2–11 children as a control. They received treatment with PVP-I 3 times at 2-month intervals for 4 months. PVP-I significantly ↓ *S. mutans* levels at 6 months in both groups (*p* = 0.003), with *p* = 0.0005 in Group 1 and *p* = 0.004 in Group 2. At 1-year follow-up, 2 experimental children and 5 from the control presented caries, however, the difference was not statistically significant (*p* = 0.06). This suggests PVP-I’s potential role in dental caries prevention, however, more clinical studies are needed to confirm this hypothesis.	[157]
Pre-procedural rinse	1% PVP-I mouthwash	In vivo	SARS-CoV-2	Twenty-five patients gargled with PVP-I for 30 s. After 5 min, the saliva was collected, and it was observed that the mouthwash significantly ↓ the viral load in the mouth cavity compared to 0 subjects from the control (*p* < 0.0001). This demonstrated that PVP-I mouthwash can decrease the spread of SARS-CoV-2 during dental treatments.	[138]
Alveolar osteitis (dry socket)	1% Betadine mouthwash	Controlled trial	-	Ninety-seven patients rinsed preoperatively with 1% betadine mouthwash before the surgical extraction of mandibular third molars, while ninety-two subjects were the controls. There was a significant difference in the ↓ incidence of dry socket in the experimental group compared to the control (*p* = 0.036), with 5 subjects who received treatment experiencing alveolar osteitis compared to 13 in the control. Additionally, it was demonstrated that the incidence of alveolar osteitis was correlated with an increase in age (*p* = 0.003). People older than 30 years presented a higher incidence of dry socket.	[139]

Abbreviations: ↓ = reduced; s = seconds; min = minutes; *p* < 0.05 = significant differences; *p* < 0.05 = no significant differences.

**Table 4:** Supporting evidence for NaOCl usage.

Uses	Formulation	Type of Study	Spectrum	Results	Ref
Dental caries	2.25% NaOCl gel	Controlled clinical trial	-	Ten children received NaOCl gel for 2 min. The application was repeated until caries-free. It was effective in removing carious dentine from primary teeth, although conventional methods such as drilling are considered to be faster (*p* < 0.001). Additionally, the treatment lowered the pain score compared to conventional treatment (*p* = 0.005).	[177]
Gingivitis	0.2% NaOCl mouthwash	Controlled clinical trial	*Streptococcus* spp., *Enterococcus faecalis*	Sixty patients were divided into two equal groups and received either 0.2% CHX mouthwash or 0.2% NaOCl mouthwash. In both groups, there was a significant difference from baseline and by day 21 in the plaque index, gingival index, and gingival bleeding index. It can be an alternative to 0.2% CHX mouthwash for removing debris and reducing the incidence of plaque-induced gingivitis due to better tolerability.	[180]
Biofilm reduction	Mixture of an aminoacid (AA) and 0.475%, 0.2%, 0.3%, 0.4% NaOCl gel	In vitro	*Porphyromonas gingivalis*, *Tannerella forsythia*, *Fusobacterium nucleatum*, *Streptococcus gordonii*, *Actinomyces naeslundii*, *Parvimonas micra*, *Prevotella intermedia*, *Aggregatibacter actinomycetemcomitans*, *Campylobacter rectus*, *Eikenella corrodens*, *Filifactor alocis*, *Capnocytophaga gingivalis*, *Eubacterium nodatum*	All mixtures significantly ↓ CFU counts compared to the control (*p* < 0.001). However, the gel containing 0.3% NaOCl was the most active. Biofilm quantity was significantly lower at 0.2% (*p* = 0.004) and 0.3% (*p* = 0.012) concentrations of NaOCl vs control. Only the 0.4% concentration ↓ biofilm metabolic activity (*p* = 0.040). The gel was more effective against Gram-negative bacteria associated with periodontitis. While it did not eliminate multiple bacterial biofilms, it significantly ↓ the number of pathogens.	[197]
Root canal treatment	1% NaOCl solution	Controlled clinical trial	*Streptococcus* spp., *Enterococcus faecalis*	After irrigation with NaOCl in 20 patients, it was observed that 65% of the canals were free of bacteria. This confirms the solution is a beneficial irrigator for treating teeth with apical periodontitis.	[178]
Yeast infection	0.525%, 5.25% NaOCl solution	In vitro	*Candida albicans*	Both formulations eliminated almost all *C. albicans* colonies from the 20 samples of silicone impressions at different time stamps. The mean ± SD: NaOCl at 0.525% after 5 min—67.55 ± 35.55; NaOCl at 0.525% after 10 min— 0 ± 0.000; 5.25% NaOCl after 5 min—0 ± 0.000; 5.25% NaOCl after 10 min—0 ± 0.000. The most potent concentration was NaOCl at 5.25%, as it ↓ *C. albicans* completely at both intervals.	[179]

Abbreviations: ↓ = reduced; CFU = colony-forming unit; SD = standard deviation; *p* ≤ 0.005 = significant differences.

**Table 5:** Supported evidence of CPC usage.

Uses	Formulation	Type of Study	Spectrum	Results	Ref.
Dental plaque reduction and gingival inflammation	0.05% CPC mouthwash	Randomized controlled trial	-	After 6 weeks of treatment, CPC mouthwash ↓ plaque and gingivitis, with fewer adverse effects, such as teeth staining, taste alteration, and numbness, compared to 0.12% CHX mouthwash.	[221]
Subgingival biofilm reduction	0.075% CPC mouthwash	In vitro	*Actinomyces naeslundii*, *Actinomyces oris*, *Actinomyces gerencseriae*, *Actinomyces israelii*, *Actinomyces odontolyticus*, *Aggregatibacter actinomycetemcomitans*, *Veillonella parvula*, *Streptococcus sanguinis*, *Streptococcus oralis*, *Staphylococcus intermedius*, *Streptococcus gordonii*, *Streptococcus mitis*, *Streptococcus constellatus*, *Streptococcus anginosus*, *Selenomonas noxia*, *Capnocytophaga ochracea*, *Capnocytophaga gingivalis*, *Eikenella corrodens*, *Capnocytophaga*, *Fusobacterium nucleatum vicentii*, *Fusobacterium nucleatum polymorphum*, *Eubacterium nodatum*, *Parvimonas micra*, *Campylobacter showae*, *Fusobacterium periodonticum*, *Prevotella intermedia*, *Porphyromonas gingivalis*, *Tannerella forsythia*, *Exiguobacterium saburrum*, *Propionibacterium acnes*, *Gemella marbillorum*	There were 8 treatments in total with a twice-daily application, with 1 min exposure per application. It decreased the biofilm’s metabolic activity by 60% and ↓ the bacterial total counts by 96%. The only remaining pathogens from the multispecies biofilm were *F.n. vincentii*, and *P. gingivalis.* This attests to the potential benefits of CPC as an adjuvant periodontal treatment.	[233]
Dental implant surgery	0.07% CPC mouthwash	Randomized controlled clinical trial	*Porphyromonas gingivalis*, *Prevotella intermedia*, *Tannerella forsythia*, *Treponema denticola*, *Aggregatibacter actinomycetemcomitans*	Patients rinsed with 15 mL of the CPC mouthwash for 60 s preoperatively. This treatment ↓ all pathogen levels, except for *A. actinomycetemcomitans.*	[234]
Viral infections	0.05%, 0.07% CPC mouthwashes	In vitro	Herpes simplex virus Type 1(HSV-1), Human papillomavirus (HPV)	CPC mouthwashes had virucidal activity on HSV-1 after 1–2 min of application.	[220]

Abbreviations: ↓ = reduced; s = seconds.

**Table 6:** Supporting evidence for HP usage.

Uses	Formulation	Type of Study	Spectrum	Results	Ref.
Biofilm reduction on dental implants	3% H_2_O_2_ solution	In vitro	*Candida albicans*, *Staphylococcus aureus*	It ↓ both fungal and bacterial numbers, making it a potential disinfectant agent for peri-implantitis.	[247]
Periodontal therapy	3% H_2_O_2_ solution	Clinical trial	-	It ↓ gingival bleeding, lowered potential inflammation, and ↓ probing depths, making it a subgingival irrigant suitable for periodontal treatment.	[253]
Gingivitis	0.8% H_2_O_2_ mouthwash	Clinical trial	-	It ↓ the plaque index that can contribute to or aggravate gingivitis.	[254]
Halitosis	H_2_O_2_ solution	Randomized controlled study	-	It ↓ bacterial counts from the tongue’s surface, which is the primary cause of halitosis.	[256]
COVID-19	1% H_2_O_2_ mouthrinse		SARS-CoV-2	It ↓ the viral load in 1 min, effectively preventing potential infection from spreading in dental cabinets.	[269]
Teeth whitening	6% H_2_O_2_ paint-on varnish, 6% H_2_O_2_ tray	Randomized double-blinded clinical trial	-	Both formulations properly bleached the teeth of the 40 patients and maintained a whiter appearance up to the 6-month follow-up with significant differences in color from the baseline (*p* < 0.05)	[260]

Abbreviations: ↓ = reduced; *p* < 0.05 = significant differences.

**Table 7:** Adverse effects of the synthetic compounds and differences in severity and frequency.

	CHX [21,68,69,70,71,72,73,74,75]	OCT [86,99,125]	PVP-I [133,134,146,147,162,281,282,283]	NaOCl [198,199]	CPC [213,236,237]	H_2_O_2_ [273,274,275,284]
Frequent	Teeth and tongue staining, unpleasant taste, xerostomia, teeth, and tongue discoloration	Teeth and tongue discoloration and dysgeusia	-	Unpleasant taste	Mouth ulcerations	Tooth sensitivity, gingival irritation, and oral lesions
Not frequent	Burning sensation, taste alteration, irritation, tongue numbness, and changes in oral mucosa	Teeth and tongue staining, burning sensations, ulcerations, irritation, nausea, and headaches	Staining of teeth and tongue, unpleasant taste, and rise in TSH levels	Tooth staining, redness of tongue, burning sensation, and xerostomia	Teeth staining, tongue numbness, taste disturbances, gingival bleeding, and sublingual swelling	-
Rare	Allergic reactions	Anaphylaxis	Thyrotoxicosis, seizures, and irritation	Changes in taste, mucosal irritation	Tooth sensitivity, oral discomfort, glossodynia	-
Severe	Anaphylaxis	Anaphylaxis	Thyrotoxicosis and seizures	Unpleasant taste	-	Tooth sensitivity
Moderate	Skin rashes, itching, and swelling	Dysgeusia, tongue discoloration, and headache	-	Teeth staining, xerostomia	-	-
Mild	Skin rashes, itching, and swelling	Tongue staining, dysgeusia, and tingling of the tongue	Staining of teeth and tongue, and rise in TSH levels	Teeth staining and xerostomia	Teeth and tongue staining	Tooth sensitivity and oral lesions

## Data Availability

The original contributions presented in the current study are included in the article, further inquiries can be directed to the corresponding author.
